# Adipokines in the Crosstalk between Adipose Tissues and Other Organs: Implications in Cardiometabolic Diseases

**DOI:** 10.3390/biomedicines12092129

**Published:** 2024-09-19

**Authors:** Shaghayegh Hemat Jouy, Sukrutha Mohan, Giorgia Scichilone, Amro Mostafa, Abeer M. Mahmoud

**Affiliations:** 1 Department of Exercise Physiology, Faculty of Physical Education and Sport Sciences, Central Tehran Branch, Islamic Azad University, Tehran 14778-93855, Iran; shaghayegh.hematjouy@gmail.com; 2 Department of Medicine, Division of Endocrinology, Diabetes, and Metabolism, College of Medicine, University of Illinois Chicago, Chicago, IL 60612, USA; smohan34@uic.edu (S.M.); gscic@uic.edu (G.S.); 3 Department of Pharmacology, College of Medicine, University of Illinois Chicago, Chicago, IL 60612, USA; amost2@uic.edu; 4 Department of Kinesiology and Nutrition, College of Applied Health Sciences, University of Illinois Chicago, Chicago, IL 60612, USA

**Keywords:** adipose tissue, adipokines, crosstalk, obesity, diabetes, cardiovascular, metabolism, inflammation, insulin resistance

## Abstract

Adipose tissue was previously regarded as a dormant organ for lipid storage until the identification of adiponectin and leptin in the early 1990s. This revelation unveiled the dynamic endocrine function of adipose tissue, which has expanded further. Adipose tissue has emerged in recent decades as a multifunctional organ that plays a significant role in energy metabolism and homeostasis. Currently, it is evident that adipose tissue primarily performs its function by secreting a diverse array of signaling molecules known as adipokines. Apart from their pivotal function in energy expenditure and metabolism regulation, these adipokines exert significant influence over a multitude of biological processes, including but not limited to inflammation, thermoregulation, immune response, vascular function, and insulin sensitivity. Adipokines are pivotal in regulating numerous biological processes within adipose tissue and facilitating communication between adipose tissue and various organs, including the brain, gut, pancreas, endothelial cells, liver, muscle, and more. Dysregulated adipokines have been implicated in several metabolic diseases, like obesity and diabetes, as well as cardiovascular diseases. In this article, we attempted to describe the significance of adipokines in developing metabolic and cardiovascular diseases and highlight their role in the crosstalk between adipose tissues and other tissues and organs.

## 1. Introduction

The importance of adipose tissue, an organ with endocrine, autocrine, and paracrine functions, has grown substantially. In addition to adipocytes, adipose tissue contains endothelial cells, fibroblasts, pericytes, preadipocytes, and various immune cell types. These non-adipocytic cell types, also known as the stromal vascular fraction, contribute to the adipose tissue secretory function [1]. Adipose tissue is regarded as a highly active endocrine tissue due to its secretory nature. Adipose tissue secretions impact the reactions of numerous tissues, such as the hypothalamus, endothelium, kidneys, skeletal muscle, pancreas, liver, and the immune system [2]. Adipose tissue releases hormones that have cytokine-like properties, known as adipokines [3]. One of the critical roles of these adipokines is the regulation of inflammatory processes in the body. Adipokines such as leptin, resistin, visfatin, and chemerin have pro-inflammatory effects, and on the other hand, adipokines such as adiponectin, vespin, apelin, omentin, and isthmin-1 have anti-inflammatory effects [4]. In addition to their role in inflammation, adipokines regulate food intake, body weight, insulin sensitivity, immune responses, and the reproductive axis to a significant degree [5]. Adipokines also affect the cardiovascular system and some aspects of the brain, especially those related to mood, cognition, reward system, and eating behavior [6]. Apparently, adipokines play a significant role in the communication between adipose tissues and other organs and tissues. Therefore, disturbance in their secretion can impact the function of different body organs [7].

The type and quantity of various adipokines are strictly regulated; nonetheless, dysregulation of their secretion can occur under conditions such as obesity and metabolic diseases. Imbalances in adipokines affect immune system function, redox homeostasis, energy metabolism, insulin sensitivity, and various other biological functions. Alterations in these biological mechanisms initiate the development of several diseases, such as diabetes, hypertension, atherosclerosis, fatty liver disease, dementia, obstructive sleep apnea, and various kinds of cancer. [8]. An example of altered adipokines associated with obesity includes an increase in leptin and resistin levels, along with a decrease in adiponectin levels, which altogether predisposes obese individuals to develop cardiometabolic diseases, nonalcoholic fatty liver disease (NAFLD), polycystic ovary syndrome (PCOS), and autoimmune diseases [5,9].

In light of the diversity of adipocytes, this review article begins by describing the various types and distinctions of adipocytes. We then address the function of adipokines secreted by adipose tissue, focusing on their involvement in developing cardiometabolic diseases. Finally, the reciprocal influences of adipose tissue on a multitude of tissues, such as the immune system, brain, blood vessels, pancreas, liver, muscle, and gastrointestinal tract, have been addressed.

## 2. Types of the Adipose Tissue

**Figure 1:**
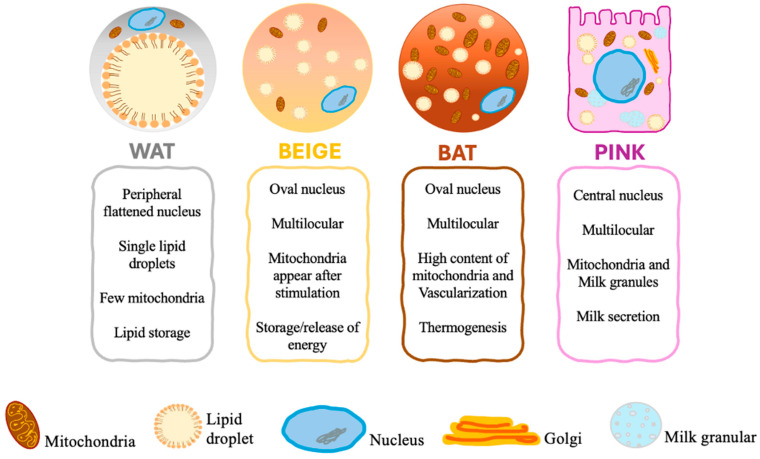
Different types of adipocytes.

There are four distinct forms of adipose tissue (Figure 1), including white adipose tissue (WAT), brown adipose tissue (BAT), beige adipose tissue, and pink adipose tissue, which play a crucial role in maintaining homeostasis [10].

WAT is present in both the subcutaneous and visceral areas. The subcutaneous adipose tissue acts as a protective barrier against the spread of infection, an insulating layer to mitigate heat dissipation, and a buffering mechanism to safeguard against mechanical strain. On the other hand, visceral adipose tissue plays a crucial role in protecting essential organs, including those in the mediastinal, epicardial, mesenteric, and omental regions. The primary role of WAT is to regulate and sustain energy balance. Energy homeostasis requires a delicate equilibrium between lipogenesis (the synthesis of lipids) and lipolysis (the breakdown of lipids), the synthesis and secretion of hormones and other soluble mediators, and the responsiveness to stimulatory and inhibitory signals facilitated by the sympathetic nervous system. Adipose tissue is critical in maintaining energy balance by storing extra energy as triglycerides in times of surplus and releasing fatty acids when energy is scarce. Lipoprotein lipases, connected to the vascular endothelium, break down triglycerides that are transported by chylomicrons and extremely low-density lipoproteins into non-esterified fatty acids (NEFA) and monoacylglycerol that are either utilized as a source of energy or stored in the WAT in the form of triglycerides [11].

Brown adipose tissue (BAT) is a particular type of fat located in the supraclavicular region, between the shoulder blades, around the kidneys, and along the spinal cord. The brown color of this fat depot is due to its profuse vascularization and high content of mitochondria [11]. Brown adipocytes differ in size, shape, and intracellular architecture. Unlike white adipocytes originating from multipotent mesenchymal stem cells, brown adipocytes originate from myogenic Myogenic Factor 5 (*Myf5*) *+* Paired Box 7 (*pax7*) *+* precursor cells and are the primary site of non-shivering thermogenesis [12]. BAT is a thermoregulatory organ that utilizes glucose and lipids as fuels for heat production to protect the body against cold exposure and maintain body weight [13]. Developmental depots of BAT are predominantly observed in infants and small mammals and aid in cold temperature management [2,14].

Brown adipocytes utilize fatty acids and glucose to generate heat in response to various stimuli. This thermogenesis is catalyzed by the Uncoupling Protein 1 (UCP1) protein, which dissipates energy by releasing protons from the mitochondrial membrane, decoupling oxidative respiration from ATP synthesis, and emitting heat [14,15]. During cold exposure, UCP-1 within the brown adipocytes oxidizes long-chain fatty acids and carbohydrates to produce heat to meet the energy demand surge [16]. The transforming growth factor (TGF)—β family of proteins, bone morphogenic factor (BMP)-7, and myostatin regulate the differentiation of brown adipocyte precursors to brown adipocytes [17,18]. The key transcription factors involved in differentiating brown adipocytes are CCAAT/enhancer-binding protein β (C/EBP-β), Forkhead box protein C2 (FOXC2), and PR domain containing 16 (PRDM16) [12,19,20]. In the myogenic lineage *Myf5 +* precursor cells, the expression of peroxisome proliferator-activated receptor gamma coactivator-1 alpha (PGC-1α) is induced by C/EBP-β and PRDM16 [21]. Subsequently, PGC-1α activates the BAT differentiation, and along with peroxisome proliferator-activated receptor gamma (PPAR-γ) and PPAR-α, it regulates mitochondrial biogenesis and energy metabolism [22].

Prolonged exposure to cold triggers beta-adrenergic activation, leading to the formation of brown fat cells within WAT stores [23]. These de novo-developed brown adipocytes within WAT are called brite (brown in white) or beige adipocytes. In fact, Beige adipocytes are a subpopulation of white adipocytes that exhibit characteristics of brown adipocytes [5,11]. Even though beige adipocytes do not develop from *Myf5 +* precursor cells and possess unique gene signatures [24], morphologically and functionally, they are similar to classic BAT [25]. Beiging occurs in humans and rodents but is much more pronounced in mice [5]. Beige adipocytes were initially implicated in the response to cold temperatures. However, recent studies have associated brown/beige adipose tissue with protection against hyperglycemia, hyperlipidemia, and obesity due to its elevated energy expenditure capacity by utilizing glucose and lipids [26]. Recent studies have also identified several factors that induce the “browning” or “beiging” of WAT. These include physical activity, nutrition, pharmaceutical agents, pre- and post-biotics, and adipokines [27].

Beige and brown adipocytes are morphologically and biochemically similar; they both contain microscopic lipid droplets, hence the term multilocular. In addition, they are packed with mitochondria and express thermogenic genes such as *UCP1*, cell death-inducing DFFA-like effector A (CIDEA), peroxisome proliferator-activated receptor (PPARα), and PGC-1α. They are also capable of thermogenesis in response to cold weather. Nevertheless, these two types of adipocytes have functional differences. Brown adipocytes have large mitochondria and express elevated amounts of UCP1 and other thermogenic elements. On the other hand, beige adipocytes require stimulation to acquire a brown adipocyte-like phenotype, and depending on environmental or physiological conditions, they either store or release energy [14].

The fourth group is pink adipocytes, which are milk-secreting adipocytes that originate as a result of the transdifferentiation of subcutaneous white adipocytes during pregnancy and lactation [5,28]. Pink adipocytes are characterized by abundant cytoplasmic lipid droplets, a round and large nucleus—central in location—apical surface with the microvilli Golgi complex, a rough endoplasmic reticulum, and milk granules. These adipocytes were designated as “pink” due to the change in their color during pregnancy and lactation. As a consequence of the absence of adipogenic transcription factors, recent research has linked pink adipocytes to the progression and development of breast cancer, as well as the establishment of a pro-tumorigenic environment [28].

Apart from the above categories of adipose tissue, which are determined mainly by the structure and function of adipocytes, adipose tissue is also classified according to its anatomical position in the body as subcutaneous and visceral. These fat depots vary in anatomical position, physiological role, and influence on general well-being. Although both forms of fat serve as energy storage, they have unique functions and impacts on metabolic and cardiovascular physiology [29]. The subcutaneous fat is located below the skin, mainly in regions such as the abdominal cavity, thighs, gluteal muscles, and upper extremities. It functions as a reservoir of energy, provides thermal insulation for the body, and offers cushioning for the skin and muscles. Subcutaneous fat typically exhibits lower metabolic activity in comparison to visceral fat. This fat synthesizes adipokines, namely leptin and adiponectin, which exert advantageous effects, such as regulating hunger and enhancing insulin sensitivity [30].

Subcutaneous fat is generally less detrimental than visceral fat and may even protect in some circumstances. Greater quantities of subcutaneous fat are linked to a reduced likelihood of metabolic disorders, provided visceral fat levels are well managed [31,32]. However, visceral fat accumulates in the deep abdominal cavity, enveloping essential organs, including the liver, pancreas, intestines, and kidneys. Although also an energy reserve, visceral fat is mainly associated with metabolic control. Nevertheless, excessive amounts of visceral fat pose substantial health hazards. Highly metabolically active visceral fat generates several pro-inflammatory adipokines, such as resistin, TNF-α, and IL-6, contributing to systemic inflammation, insulin resistance, and the onset of metabolic disorders [30,33]. The presence of visceral fat is closely linked to adverse health consequences, such as the increased risk of insulin resistance, type 2 diabetes, cardiovascular disorders, and some types of cancer [31,32]. Visceral fat accumulation is a primary catalyst for metabolic syndrome, characterized by hypertension, increased blood glucose, and altered cholesterol levels [34]. Managing visceral fat is crucial in mitigating the risk of cardiometabolic disorders, such as cardiovascular disease and diabetes.

## 3. The Role of Adipokines in Cardiometabolic Diseases

Both males and females are affected by various risk factors contributing to the development of cardiometabolic disease. Among these risk factors, obesity has gained considerable attention in recent years as a growing epidemic [35]. Weight gain is influenced by various factors, including racial and ethnic disparities, psychosocial stressors, socioeconomic factors, environmental exposures, and numerous other determinants [36]. According to the Global Burden of Disease (GBD) study, the prevalence of obesity has doubled in 73 countries from 1980 to 2019, and it continues to rise in many other nations [37]. Recent data from the National Health and Nutrition Examination Survey reveal that the prevalence of obesity increased from 37.9% in 2013–2014 to 42.4% in 2018–2019. Similarly, the prevalence of class 3 obesity, defined by a body mass index (BMI) of 40 kg/m^2^ or higher, has also increased: 5.5% among non-Hispanic white men and 16.9% among non-Hispanic black women [38]. This observed rise in obesity resulted in a three-fold surge in cardiometabolic diseases and related fatalities from 1999 to 2020 [39].

**Figure 2:**
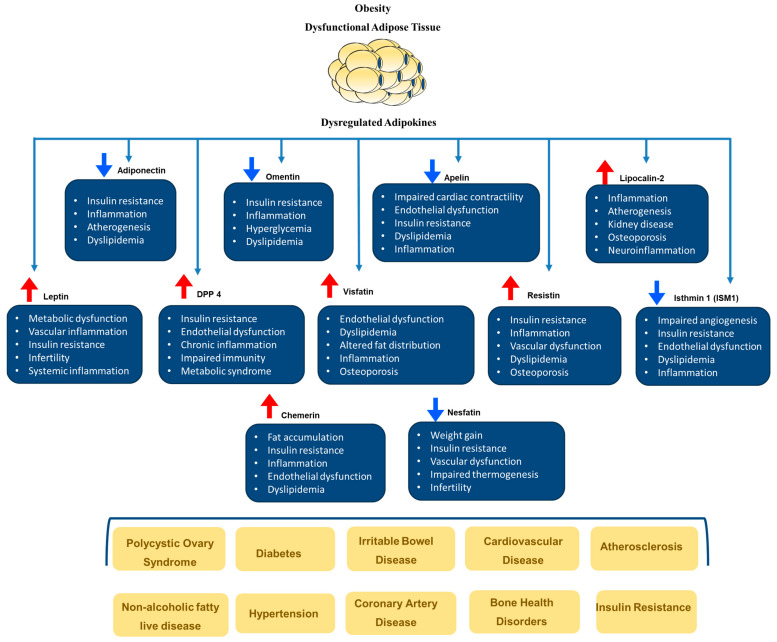
The role of dysregulated adipokines in the development of cardiometabolic diseases. Blue arrows indicate reductions, and red arrows indicate increases in adipokines.

Obesity plays a significant role in the development of cardiovascular risk factors such as hypertension, type 2 diabetes (T2DM), sleep disorders, dyslipidemia, and others. Evidence suggests that abdominal adiposity, specifically fat around the internal organs (visceral fat), predicts cardiovascular diseases (CVD). Indeed, the advancement of scanning tools such as dual-energy X-ray absorptiometry (DEXA) and bioelectrical impedance analysis (BIA) has facilitated in-depth examinations of human body composition and its relationship with CVD. Notably, even when individuals have the same BMI or body fat percentage, there can be differences in how subcutaneous and visceral fat are distributed. The phrase “metabolically healthy obesity” was created to describe individuals who are obese but have lower levels of visceral fat [36]. According to Camhi et al. [40], individuals with metabolically healthy obesity have a lower cardiometabolic risk compared to those with higher visceral fat, irrespective of their BMI. This concept was supported by research conducted by Britton and Neeland et al. [41,42], which confirmed the relationship between visceral fat and the increased incidence of dysmetabolic states, fatty liver, and CVD. Obesity is characterized by dysfunctional adipose tissue, which includes altered adipokine release, increased chronic inflammation, dysregulated lipid metabolism, and decreased vascular function. These characteristics are all associated with obesity. These alterations are significant contributors to the development of metabolic syndrome, systemic insulin resistance, and an elevated risk of cardiovascular disease. The most frequently reported health outcomes of dysregulated adipokines are depicted in Figure 2.

Soluble mediators and adipokines secreted by different fat depots can enter the bloodstream and spread throughout the body, affecting distant tissues and organs. Furthermore, specific adipose tissue depots located next to essential organs might impact the function of these organs in a paracrine manner. For example, perivascular adipose tissue has been demonstrated to affect vascular function, and epicardial adipose tissue influences myocardial performance [43]. The subsequent section will delineate several significant adipokines secreted by adipose tissue and their associations with obesity and cardiometabolic diseases. It is important to note that the adipokine profile and function are contingent upon the anatomical location of fat and its type, specifically WAT versus BAT. For example, adipokines produced by brown or beige adipocytes in subcutaneous adipose tissues, such as fibroblast growth factor 21 (FGF21), demonstrate thermogenic functions, whereas adipokines produced by white adipocytes in the same fat depots, like adiponectin and leptin, are mainly concerned with energy intake and storage. Consequently, distinct sections will be designated for WAT and BAT adipokines and the changes that occur under specific pathological conditions, such as obesity and metabolic diseases.

## 4. White Adipose Tissue (WAT) Adipokines

**Figure 3:**
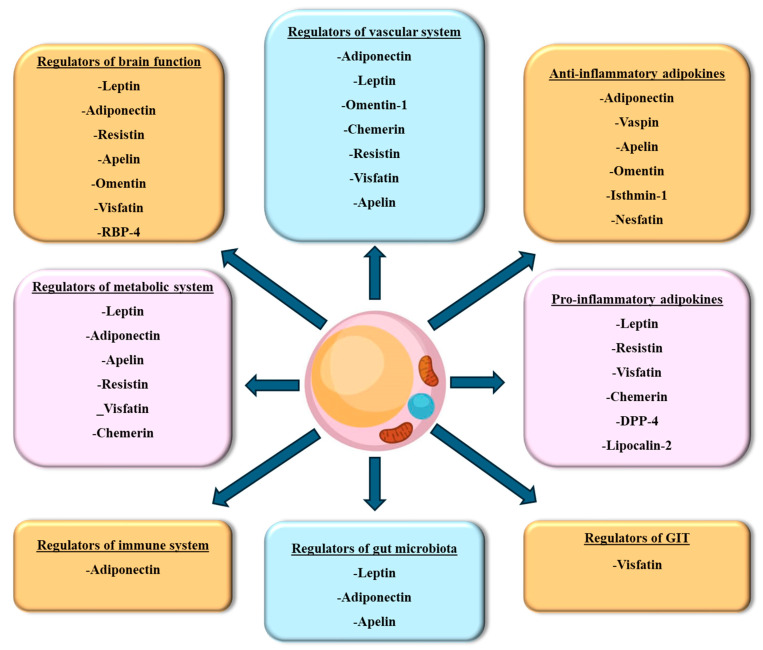
Classification of WAT secreted adipokines. Adipokines secreted from WAT are classified based on the target tissue of their effect. DPP-4, dipeptidyl peptidase-4; RBP-4, retinol-binding protein-4.

WAT adipokines are primarily involved in energy storage and homeostasis. These adipokines demonstrate that WAT is not merely a passive site for fat storage but actively engages in diverse physiological functions, particularly those related to metabolic regulation, immune function, and inflammation. Certain adipokines control energy equilibrium by inhibiting appetite, which in turn aids in regulating body weight. Other adipokines regulate blood coagulation homeostasis, suppressing inflammation or improving insulin sensitivity. Conversely, certain WAT adipokines exhibit pro-inflammatory properties and can exacerbate insulin resistance, mainly when produced in excess. The subsequent section delves into the WAT adipokines identified thus far and their physiological role that, when dysregulated, results in the development of cardiometabolic diseases (Figure 3).

### 4.1. Leptin

Leptin is a hormone that consists of 146 amino acids and is synthesized by WAT and coded by the obese (*LEP* or *ob*) gene on chromosome 7q31.3. Leptin shares structural similarities with pro-inflammatory cytokines and acts by binding to its receptor, leptin receptors (LR), which can be found on the cell surface of various tissues, including neurons, pancreatic cells, cardiac cells, hepatic cells, and intestinal tissues [44]. Leptin receptors are members of the cytokine receptor family and comprise six isoforms, with isoform-b being the most prominent member. The leptin receptor dimerizes upon leptin binding, activating the Janus kinase 2 (JAK)/signal transducer and activator of transcription (STAT) signaling pathway and binding proteins such as Src homology phosphatase-2 (SHP2), STAT5, and STAT3, facilitating downstream signaling events [44]. Leptin has gained considerable clinical significance due to the multitude of effects resulting from its increased or decreased levels. These effects range from heightened vulnerability to infections to the development of autoimmune disorders. The growing understanding of leptin’s impact has emphasized its significance in clinical practice [44]. Furthermore, leptin’s angiogenic and atherogenic effects have led to its recognition as an essential marker in obesity, diabetes, and CVD [1].

Leptin is involved in regulating glucose balance at the peripheral and central levels. It exerts various metabolic effects, including suppressing the production of glucagon and corticosterone, increasing glucose uptake, and inhibiting hepatic glucose output [45]. Centrally, leptin primarily acts on the brainstem, particularly the solitary tract and ventral tegmental area, as well as specific portions of the hypothalamus, including the lateral and ventromedial hypothalamic (VMH) areas, dorsomedial, ventral premammillary, and arcuate nuclei (ARC). In the medulla, leptin regulates satiety and controls reward and aversion. The functions of leptin in the VMH and ARC have been implicated in glucose regulation. The ARC nucleus is vital in the regulation of appetite and energy balance. It consists of neurons containing two peptides: orexigenic agouti-related protein/neuropeptide Y (AgRP/NPY) and anorexigenic proopiomelanocortin (POMC). Leptin stimulates POMC neurons, which promote satiety and appetite suppression, while inhibiting AgRP/NPY neurons, which stimulate appetite [44]. GABAergic neurons, especially AgRP neurons, are necessary and sufficient for leptin’s glucose-lowering effects. The melanocortin-4 receptor (MC4R), which mediates the downstream effects of alpha-melanocyte-stimulating hormone (α-MSH) and AgRP, also contributes to leptin’s glucose-lowering actions [45].

In addition to its central action, leptin directly regulates glucose in several metabolic tissues and regulates the secretion of hormones from the endocrine pancreas. Leptin has been shown to reduce insulin secretion in humans, preclinical models, and in vitro cultured beta cells. The mechanism behind this involves activating and translocating ATP-dependent K^+^ channels to the cell membrane, which hyperpolarizes the beta cell membrane and consequently decreases insulin secretion [46]. Leptin therapy also lowers circulating glucagon levels through mechanisms that involve the central nervous system (CNS) and possibly leptin receptors on alpha cells [45]. In vital metabolic tissues such as skeletal muscle, leptin is essential for preserving glucose balance by improving glucose absorption, glycogen formation, and the oxidation of glucose and fatty acids [47]. These effects were mediated via leptin-induced 5′-adenosine monophosphate-activated protein kinase (AMPK) activity. In adipose tissues, leptin counteracts insulin’s action [47] by inhibiting insulin receptor kinase activity, reducing insulin-induced IRS-1 activation, and binding to PI3K [48]. Nevertheless, leptin increases glucose uptake and energy expenditure in adipose tissues through its central action, independent of insulin’s action. Leptin also maintains glucose homeostasis by upregulating lipolysis when insulin is high and downregulating it when insulin is low. This effect maintains low glycerol and fatty acid levels, suppressing gluconeogenesis [45]. Within the liver, leptin directly inhibits gluconeogenesis and glycogenolysis in hepatocytes, culminating in an overall enhancement of insulin-mediated inhibition of hepatic glucose production [45].

Leptin is crucial in maintaining a balance between food intake and energy expenditure. When adipose tissue decreases, leptin levels decrease as well. As a result, the amount of leptin that crosses the blood–brain barrier is reduced, signaling an energy deficit that subsequently increases the appetite and enhances food consumption. This, in turn, stimulates leptin production to counteract the energy deficit and restore the balance in a tightly regulated feedback mechanism [44]. Hyperleptinemia, on the other hand, is a condition where leptin is produced in excess due to peripheral leptin resistance and is associated with obesity, metabolic diseases, and increased cardiovascular risk.

Leptin resistance, a condition in which leptin’s capacity to control hunger and maintain body weight is impaired, has been detected in obese people and is linked to hyperleptinemia. In obese individuals, there is a direct correlation between leptin levels and the percentage of body fat. Additionally, there is an increase in *LEP* gene expression in adipose tissues compared to lean individuals. The causes of leptin resistance are hypothesized to include impaired transport of leptin across the blood–brain barrier or malfunctioning intracellular signaling mechanisms of the leptin receptor. Hyperleptinemia is associated with NAFLD, neurodegenerative disorders, eating disorders, and several other diseases [44]. Research conducted by Yadav and Muskiet et al. [49,50] has demonstrated a positive association between leptin levels and various anthropometric measures such as insulin resistance, waist circumference, BMI, and hip circumference [49,50]. A case–control study involving 87 obese individuals with T2DM and 85 healthy individuals revealed higher leptin levels in obese individuals, especially in severely obese females, compared to the healthy group [51]. In this study, elevated leptin levels have been linked to the development of atherosclerosis and have been identified as an independent predictor of carotid intima-media thickness (cIMT). Additionally, leptin was found to act as a chemoattractant and migration promoter for monocytes and macrophages, further accelerating the progression of atherosclerosis [1]. This process is accompanied by an increase in the production of reactive oxygen species, contributing to the development and advancement of atherosclerosis.

Elevated leptin levels contribute to vascular dysfunction via downregulating PPARγ, a protein that regulates vascular tone via promoting nitric oxide production. Leptin also induces the proliferation, migration, and calcification of vascular smooth muscle cells (VSMCs), contributing to the progression of atherosclerosis [1]. In addition, the pro-thrombotic effects of leptin summarized in a review article by Su et al. [52] include the action of leptin in stimulating platelet aggregation while inhibiting fibrinolysis and coagulation. Several studies conducted on mice lacking leptin receptors and exhibiting hyperliptinemia demonstrated thrombotic vascular occlusion and an interaction between leptin and platelet receptors, further supporting the involvement of leptin in thrombotic processes [52]. In support of these studies, Dellas et al. [53] have shown that leptin activates human platelets, leading to an increased expression of integrin αIIbβ3 and adhesion of platelets to both soluble and immobilized fibrinogen. Others have also reported leptin-dependent aggregation of human platelets mediated by phospholipase A2, protein kinase C, and phospholipase Cγ2 [54].

Apart from leptin resistance, congenital lack of leptin, resulting from mutations in the *LEP* gene, has additionally been associated with extreme obesity, excessive appetite, continual search for food, reduced feeling of fullness, repeated bacterial infections, hepatic steatosis, dyslipidemia, hyperinsulinemia, and hypogonadotropic hypogonadism [44]. Patients with this condition benefit from leptin administration, which has demonstrated therapeutic effects on various aspects such as glucose metabolism, energy expenditure, body weight, dietary intake, and lipid metabolism [3].

### 4.2. Adiponectin

Adiponectin is a protein consisting of 224 amino acids and is primarily synthesized and secreted by WAT. The gene encoding adiponectin is located on chromosome 3q27 [55]. Adiponectin is known for its significantly elevated levels in the bloodstream, around 1000 times greater than other adipokines. However, in obese individuals, adiponectin levels tend to be lower compared to non-obese individuals. Adiponectin is present in different forms, including low-molecular-weight (LMW), medium-molecular-weight (MMW), high-molecular-weight (HMW), and globular adiponectin. Among these forms, MMW and HMW oligomers are the predominant ones in humans, while the LMW trimer comprises less than 30% of the total adiponectin. Notably, the HMW form of adiponectin is mainly associated with decreased glucose levels and increased insulin sensitivity [5].

The signaling pathways of adiponectin are primarily mediated by two key receptors, AdipoR1 and AdipoR2. Both receptors are members of the seven-transmembrane domain receptor family, structurally comparable to G-protein-coupled receptors (GPCRs) [56]. However, they do not possess the unique G-protein binding domains characteristic of GPCRs. AdipoR receptors exhibit an atypical structure in comparison to the other seven transmembrane receptors, whereby their N-terminal is located within the cytoplasm while their C-terminal is situated outside the cell. Each receptor possesses a binding pocket that specifically interacts with adiponectin [57]. This structure enables selective interaction with the globular and full-length forms of adiponectin, which in turn activate subcellular signaling pathways such as AMP-activated protein kinase (AMPK) and peroxisome proliferator-activated receptor-α (PPAR-α) [58]. Despite their substantial structural resemblances, AdipoR1 and AdipoR2 vary in their distribution throughout tissues and their downstream effects. These variations contribute to their unique functions in regulating metabolic processes [59]. The principal function of AdipoR1 is to bind the globular form of adiponectin, therefore promoting the activity of AMP-activated protein kinase (AMPK) and facilitating the absorption of glucose and oxidation of fatty acids. AdipoR1 is highly prevalent in skeletal muscle and is crucial in maintaining glucose balance and cellular energy expenditure via stimulating AMPK [60]. The globular form of adiponectin and AdipoR1 play a vital role in controlling AMPK activation in response to physical activity, contributing significantly to exercise-induced improvements in metabolic health [61]. AdipoR2 exhibits greater selectivity for full-length adiponectin, which controls glucose and lipid metabolism by activating PPAR-α. The liver is the primary site of AdipoR2 expression, where it plays a crucial role in regulating fatty acid metabolism and insulin sensitivity. It achieves this by facilitating the oxidation of fatty acids and decreasing hepatic glucose synthesis. The adipoR2 receptor is crucial in reducing triglyceride levels, decreasing liver fat accumulation, and enhancing overall lipid profiles, thereby protecting against metabolic diseases such as non-alcoholic fatty liver disease (NAFLD) [62]. Dysfunctional adiponectin signaling via AdipoR1 and AdipoR2 is linked to the development of insulin resistance, obesity, and type 2 diabetes. Impairment of adiponectin action and deterioration of cardiometabolic health have been attributed to decreased expression or activity of these receptors in persons with metabolic syndrome [63]. In summary, AdipoR1 and AdipoR2 are receptors for adiponectin that have structural similarities but demonstrate functional differences. Collectively, these receptors play a vital role in preserving metabolic balance and are promising targets for treating chronic cardiometabolic disorders.

Adiponectin exhibits various beneficial properties, such as anti-inflammatory, anti-diabetic, and anti-atherosclerotic effects. Adiponectin plays a role in increasing insulin sensitivity, which helps improve the body’s response to insulin. Additionally, it promotes fatty acid oxidation, leading to a reduction in hepatic glucose production. These actions contribute to the overall lipid and glucose metabolism regulation, making adiponectin a critical factor in maintaining metabolic health [1]. Adiponectin interacts with its receptors, AdipoR1 and AdipoR2, to initiate a cascade of events, activating AMPK and endothelial nitric oxide synthase (eNOS), enhancing vascular functions. Adiponectin also has anti-inflammatory properties; it inhibits TNF-α, suppresses inflammatory cytokines, activates NF-κB, and influences the proliferation and migration of VSMCs. Additionally, adiponectin induces macrophage polarization to the anti-inflammatory M2 subtype while decreasing the pro-inflammatory M1 macrophages and reducing inflammation [1].

Reduced adiponectin levels were linked to an increased likelihood of developing CVD [1]. A study conducted by Gradinaru et al. [54] to investigate the connections between adiponectin and cardiovascular risk factors in elderly patients with metabolic syndrome demonstrated significant positive correlations between adiponectin and high-density lipoprotein (HDL) (*p* < 0.05), the total cholesterol/low-density lipoproteins (LDL) ratio (*p* < 0.01), and improved endothelial function. Furthermore, this study revealed an inverse relationship between adiponectin and the homeostasis model assessment of insulin resistance (HOMA-IR; r = −0.348; *p* < 0.05) and serum lipid peroxidation (*r* = −0.037; *p* < 0.05) while showing a direct association with antioxidant capacity (*r* = 0.339; *p* < 0.05). Finally, a negative relationship was noted between adiponectin levels and cIMT, a marker of atherosclerosis. This study and several others have provided detailed insights into the protective effects of adiponectin and confirmed the association between low adiponectin concentrations in obesity and CVD, like coronary artery disease, ventricular dysfunction, myocardial infarction, atherosclerosis, hypertension, and others [64].

Although elevated adiponectin levels are typically seen as advantageous for metabolic health, there is a U-shaped correlation between adiponectin levels and health outcomes, where both low and overly high adiponectin levels have been linked to different disease states [65,66]. Obesity, insulin resistance, and metabolic disorders are frequently linked to reduced levels of adiponectin. Insufficient adiponectin levels hinder the responsiveness of tissues such as muscle and liver to insulin, inhibiting their capacity to absorb glucose [67]. This phenomenon exacerbates hyperglycemia and accelerates the development of insulin resistance [68]. Insufficient adiponectin levels are associated with higher fat deposition, visceral fat, and impaired metabolic function [30]. An increased risk of coronary artery disease, hypertension, and myocardial infarction is associated with low adiponectin levels, primarily because of its diminished anti-inflammatory and anti-atherogenic properties [69]. Additionally, low adiponectin levels elevate the likelihood of liver steatosis and inflammation, which are major causes of non-alcoholic fatty liver disease (NAFLD) [70].

On the other hand, too elevated amounts of adiponectin can have disadvantageous effects. Advanced heart failure commonly presents with increased levels of adiponectin, which might serve as a compensatory response to metabolic load or cardiac dysfunction. However, it can also suggest worsening of the condition [71,72]. Furthermore, adiponectin levels were demonstrated to increase in chronic kidney disease (CKD), particularly in cases of end-stage renal disease. Impairment of renal function is proposed to decrease the clearance of adiponectin, and elevated levels may indicate kidney damage rather than a direct protective mechanism [73]. Moreover, elevated plasma adiponectin levels have been associated with heightened joint inflammation in autoimmune disorders such as rheumatoid arthritis. Adiponectin plays a role in stimulating inflammatory processes in joint tissues, exacerbating symptoms of the condition [74]. Indeed, those with anorexia nervosa frequently exhibit increased levels of adiponectin, which indicates the body’s attempt to sustain energy balance in reaction to insufficient food intake [75]. Collectively, the association between low and high adiponectin levels and disease reflects the complex function of this adipokine in health and pathology. Consequently, preserving appropriately balanced adiponectin levels is essential for overall well-being and cardiometabolic health.

Based on these findings, new synthetic analogs of naturally occurring adiponectin, such as AdipoRON and AdipoAI, have been developed and demonstrated promising effects in improving blood glucose and insulin sensitivity in diet-induced obese mouse models. These analogs mimic the pharmacological effects of endogenous adiponectin and offer the possibility of similar therapeutic benefits for obese and diabetic individuals [76]. Previous preclinical investigations have demonstrated that AdipoRON can produce a similar effect to adiponectin [77]. This molecule stimulates both AdipoR1 and AdipoR2 receptors, enhancing mitochondrial activity and glucose and lipid metabolism [78,79]. Furthermore, AdipoRON has demonstrated the ability to inhibit inflammation and reduce oxidative damage [80]. Given these properties, adipoRON has emerged as the preferred medication for cardiometabolic disorders marked by inflammation and oxidative stress, such as diabetic cardiomyopathy. AdipoRon was shown to effectively decrease heart fibrosis and lipotoxicity and enhance insulin sensitivity in mouse models of diabetes [81]. These effects were attained by modifying inflammation-related pathways, namely Toll-like receptor 4 (TLR4). Cultured human cardiomyocytes preconditioned with high glucose levels, which serve as a model for diabetic cardiomyopathy, also showed that AdipoRON has an impact on lowering inflammation and oxidative stress and improving metabolic processes. These favorable outcomes were accomplished by enhancing the PPARα and AMPK pathways while inhibiting TLR4 inflammatory signals [81]. Comparable results were documented in diabetic mice and rats, where adipoRON enhanced systolic and diastolic function, left ventricular metrics, and myocardial dynamics [82,83]. A systematic review by Laurindo et al. [84] summarized several of the cardioprotective and nephroprotective effects of AdipoRON. Collectively, adiponectin receptor agonists, including AdipoRON, are prospective agents for improving cardiometabolic diseases through various mechanisms that necessitate additional research.

### 4.3. Resistin

Resistin is a small polypeptide (108 amino acids) having a molecular mass of approximately 12.5 kDa. In humans, resistin is coded by the gene *RETN* [85]. Clinical studies have demonstrated higher resistin (hyper-resistinemia) levels in individuals with T2DM and obesity. In those individuals, resistin levels were directly associated with insulin resistance, while no correlation was observed in those with normal circulating resistin levels [86]. In obesity, high resistin levels directly inhibit insulin-induced glucose uptake in adipocytes. Resistin is implicated in cardiovascular, metabolic, and autoimmune diseases, as it affects molecular pathways associated with inflammation, including the activation of NFKB1 nuclear factor kappa B (NF-kB) by Toll-like receptor 4 (TLR4). As a result of its influence on endothelial dysfunction, resistin has become increasingly significant in the progression of atherosclerosis. Resistin also plays a role in thrombosis, migration, angiogenesis, and the proliferation of VSMCs. Additionally, it affects cholesterol metabolism by modulating the PPARγ-dependent phosphatidylinositol 3-kinase (PI3K)/protein kinase B (AKT) pathway, with PPARγ ligands enhancing resistin activity. Overall, it is evident that resistin contributes to the development of obesity-related complications [1]. Currently, resistin inhibitors like masoprocol are being regarded as promising medications for treating obesity and its associated metabolic and CVDs [87].

### 4.4. Chemerin

Chemerin is a preprotein consisting of 163 amino acids, and its production is encoded by the retinoic acid receptor responder protein 2 (*RARRES2*) gene. Chemerin is primarily secreted by adipose tissue and skin as a pre-pro-chemerin, which is processed by various serine and cysteine proteases into different isoforms. Recent studies have shown the involvement of chemerin and its receptors in various physiological processes. The receptors associated with chemerin are G protein-coupled receptor 1 (GPR1), chemerin chemokine-like receptor 1 (CMKLR1), and C-C chemokine receptor-like 2 (CCRL2).

Chemerin and its receptors play roles in adipogenesis, angiogenesis, osteoclastogenesis (the formation and activation of bone-resorbing cells called osteoclasts), and inflammatory processes in the skin and adipose tissue [88]. Increased concentrations of chemerin have been detected in individuals with obesity. In chronic inflammatory conditions such as obesity and diabetes, chemerin is triggered by pro-inflammatory cytokines like interleukin-1 beta (IL-1β), tumor necrosis factor-alpha (TNF-α), and interleukin-6 (IL-6), stimulating the PI3K/Akt and mitogen-activated protein kinase (MAPK) signaling pathways. Chemerin’s effects involve upregulating the expression of adhesion molecules such as vascular cell adhesion molecule 1 (VCAM-1), intracellular adhesion molecule 1 (ICAM-1), and E-selectin. As a result, monocytes adhere to endothelial cells, inducing the progression of atherosclerosis. Chemerin correlates negatively with HDL, while it positively correlates with BMI, CRP (C-reactive protein), and HOMA-IR [1].

### 4.5. Visfatin

Visfatin is an adipokine initially thought to be predominantly produced and secreted by macrophages in visceral fat in response to inflammatory signals. However, it has been discovered that visfatin is also expressed in human leukocytes, adipocytes, hepatocytes, and muscle cells. It has a molecular mass of 52 KDa and encodes a protein containing 491 amino acids. Visfatin is also known as pre-B cell colony-enhancing factor (PBEF), a cytokine involved in lymphocyte maturation and inflammatory regulation. Additionally, visfatin is considered Nicotinamide phosphoribosyltransferase (Nampt), the crucial enzyme in the biosynthesis of nicotinamide adenine dinucleotide (NAD). The actions of visfatin are believed to be endocrine, paracrine, and autocrine in nature. Its autocrine effects notably regulate insulin sensitivity in the liver [89,90].

Visfatin’s pro-inflammatory properties activate several factors and molecules involved in inflammation, including activator protein 1, TNF-α, NF-kB, IL-6, IL-8, ICAM-1, VCAM-1, E-selectin, matrix metalloproteinase 2 (MMP-2), and MMP-3 [1]. According to the studies conducted by Dahl and Lee et al. [89,90], it has been observed that visfatin has a reciprocal relationship with TNF-α. Visfatin can elevate the levels of TNF-α, and when stimulated by TNF-α, visfatin increases its own expression, suggesting a positive feedback loop and a complex interplay between these two factors in inflammatory processes [89,90]. These observations are significant since this feedback loop contributes to the progression of atherosclerosis. Visfatin expression is associated with plaque instability, as it was elevated in individuals with symptomatic carotid atherosclerosis compared to those without symptoms. Furthermore, individuals who experienced acute myocardial infarction and had ruptured plaque showed increased levels of visfatin [90]. Visfatin stimulates the expression of vascular endothelial growth factor (VEGF) by activating specific signaling pathways, which in turn promote angiogenesis. Additionally, visfatin serves as a marker for subclinical atherosclerosis, as a clear correlation between visfatin levels and cIMT was observed [1]. The circulating visfatin levels are significantly higher in patients with metabolic syndrome than in the control group. These results indicate that visfatin could potentially function as a prognostic indicator for the onset of cardiometabolic disorders [91].

### 4.6. Dipeptidyl Peptidase 4 (DPP4)

Human DPP-4 (dipeptidyl peptidase-4) consists of 766 amino acids. It includes a brief cytoplasmic segment covering 1 to 6 amino acids, a transmembrane segment spanning 7 to 28 amino acids, and an extracellular segment spanning 29 to 766 amino acids. The extracellular domain possesses dipeptidyl peptidase activity and is responsible for the enzymatic function of DPP-4. The transmembrane domain is connected to the extracellular domain by a flexible stalk spanning 29 to 39 amino acids, and its removal results in the formation of a soluble form of DPP-4. DPP-4 is widely distributed throughout the body and can be found on the surfaces of epithelial and endothelial cells. DPP4 is primarily released by adipose tissues, predominantly from mature adipocytes in the visceral region. Within the adipose tissue, DPP4 exhibits autocrine and paracrine effects, including the stimulation of inflammation [92]. DPP4 is also expressed in many other tissues, like the liver, gut, placenta, lung, and kidney [93].

Similar to other pro-inflammatory adipokines, DPP4 levels are increased in metabolic diseases. It serves as a crucial marker for adiposity and is associated with cardiovascular complications. Inhibitors targeting DPP4 have been utilized as a therapeutic approach for treating diabetes mellitus [1]. In individuals with obesity, there is a correlation between high levels of DPP4 and several factors, including increased concentrations of macrophages in WAT, elevated levels of leptin and inflammatory cytokines, and reduced adiponectin concentrations. Sedighi et al. [94] conducted a study that demonstrated a correlation between DPP4 levels and the levels of pro-inflammatory cytokines, specifically TNF-α (tumor necrosis factor-alpha) and IL-1b (interleukin-1 beta).

In a clinical trial by Stengel et al. [95], circulating DPP4, pancreatic polypeptide, and glucagon-like peptide-1 (GLP-1) were measured in hospitalized patients with different BMI. This study showed significantly higher levels of DPP4 expression and activity in obese individuals than in those with healthy body weight (*p* < 0.05). DPP4 was found to interfere with insulin action by disrupting the phosphorylation of Akt and other molecules in the insulin signaling pathway [1]. A study by El-Alameey et al. [96] provides clinical evidence supporting the decrease in DPP4 enzyme activity in response to weight loss interventions and its correlation with the enhancement of HOMA-IR in obese individuals. The data from this study also reveals strong associations between serum DPP4 enzyme activity and factors such as BMI z-score, waist-to-hip ratio, and serum triglycerides in obese individuals. Based on these findings, it has been suggested that targeting DPP4 could be beneficial in managing obesity-related cardiometabolic diseases [96].

### 4.7. Apelin

Apelin is a polypeptide encoded by the *APLN* gene on chromosome Xq25-q26.1 [97]. It is an endogenous ligand for the apelin receptor (APJ receptor), which belongs to G protein-coupled receptors. Apelin can be released into the bloodstream by adipocytes and various other cells. Apelin has been shown to possess properties that can counteract obesity and diabetes, making it a potential target for therapeutic interventions in metabolic disorders [98]. Apelin exhibits various isoforms, such as apelin-36, apelin-13, apelin-55, apelin-17, (pGlu)apelin-13, and apelin-12. These isoforms similarly activate the APJ receptor. Apelin isoforms were found to protect against conditions like ischemia, heart failure (HF), and myocardial infarction. They promote vasodilation, enhance cardiac contractility, have antihypertensive effects, prevent cardiomyocyte injury, and enhance overall heart function.

Furthermore, APJ receptor agonists, such as BMS-986224, have been demonstrated to boost cardiac output in animal models of heart failure and cardiac hypertrophy [99]. Elabela, similar to apelin, is a newly discovered natural ligand of the APJ receptor that possesses cardioprotective effects. The *Elabela* gene encodes precursor proteins that consist of 54 amino acids. These precursor proteins undergo processing to produce different subtypes, namely ELA-32, ELA-22, ELA-21, and ELA-11 [100]. Additionally, it has been observed that Elabela is prominently secreted by human embryonic stem cells (hESCs), even though these cells do not express the APJ receptor. This expression pattern suggests that APJ may not be the exclusive receptor for Elabela, implying the existence of other receptors through which Elabela exerts its effects [101].

Similar to adiponectin, both low and high concentrations of apelin have been associated with different disorders, indicating the intricate and context-dependent ways it impacts health. Apelin provides cardiovascular protection by stimulating vasodilation, enhancing cardiac contractility, and decreasing blood pressure via the generation of nitric oxide (NO) [102]. It also serves as a crucial regulator of pulmonary vascular homeostasis, enhances glucose uptake in skeletal muscle, and promotes insulin sensitivity [103]. In addition, apelin regulates water and salt balance through its interaction with vasopressin, facilitating blood pressure management and fluid homeostasis. Accordingly, low apelin levels are associated with cardiovascular diseases, metabolic disorders, and conditions such as pulmonary arterial hypertension (PAH) [104] and chronic kidney disease (CKD) [105].

On the other hand, the elevation of apelin levels does not always result in enhanced metabolic outcomes. It can also contribute to low-grade systemic inflammation, increased insulin resistance, and adipose tissue inflammation [106]. Also, under specific circumstances, apelin may induce pro-inflammatory effects by enhancing the production of cytokines and the infiltration of immune cells [107]. High apelin levels have been shown to exacerbate inflammation and contribute to disease flare-ups in conditions such as inflammatory bowel disease (IBD) [108]. Furthermore, there is conflicting evidence regarding the role of apelin in organ fibrosis. Some studies have shown that apelin protects against liver, renal, pulmonary, and cardiac fibrosis [109]. Nevertheless, others demonstrated that high levels of apelin promote liver fibrosis via a mechanism that includes the ERK signaling pathway [110]. In summary, low and high apelin levels can be detrimental, depending on the context, and maintaining balanced apelin is crucial for optimal health.

### 4.8. Omentin

Omentin is an adipokine (38–40 kDa) initially discovered in omental adipose tissue. However, it has been found that omentin can also be produced by cells not associated with adipose tissue, like intestinal Paneth cells. Two omentin genes situated adjacently in the 1q22-q23 chromosomal region produce two variations known as omentin-1 and omentin-2. In humans, omentin-1 is the primary form found in both plasma and adipose tissue. Its synthesis is controlled by levels of glucose and insulin [111].

Omentin, another adipokine with anti-inflammatory properties, has been extensively studied in the context of obesity, diabetes, CVD, and inflammatory bowel disease. Its mechanism of action involves inhibiting the expression of inflammatory signaling pathways. This inhibition affects various factors, including mRNA, TLR4 protein, and NF-kB phosphorylation. Among the main omentin isoforms, omentin-1 has been found to promote the phosphorylation of the integrin pathway, Akt, and AMPK in macrophages. This action, in turn, inhibits the production of inflammatory cytokines. Notably, higher concentrations of omentin-1 in the blood are linked to reduced levels of inflammatory cytokines. Like adiponectin, omentin positively impacts blood vessels by increasing nitric oxide generation while decreasing apoptosis and oxidative stress [1].

Shibata et al. [112] performed a study to investigate the relationship between omentin levels and cIMT in 100 healthy Japanese men. A high-resolution carotid ultrasound was used to measure the maximum and mean cIMT in the common carotid artery. Omentin levels were assessed along with various parameters, including BMI, waist circumference, fasting glucose, creatinine, maximal cIMT, mean cIMT, and glomerular filtration rate (GFR). This study revealed negative correlations between omentin levels and BMI, waist circumference, fasting glucose, creatinine, maximal cIMT, and mean cIMT. Conversely, a direct relationship was observed between omentin levels and GFR. Based on these results, the study suggested that measuring omentin levels could be a beneficial marker of subclinical atherosclerosis and cardiometabolic risk [112].

A study conducted by Yang et al. [113] on 109 patients with atherosclerotic acute cerebral infarction (ACI) examined the correlation between omentin-1 levels at admission and the severity, functional prognosis, and infarction volume 90 days after the ACI incident. At the time of admission, patients with ACI exhibited lower levels of omentin-1 than healthy individuals, according to this study (47.18 ± 13.64 ng/mL vs. 56.27 ± 34.44 ng/mL, *p* = 0.014). Furthermore, 90 days after the infarction, high omentin-1 levels (>43.10 ng/mL) were inversely linked with an adverse functional prognosis [113]. Hence, omentin potentially serves as a significant indicator of atherosclerosis and CVD.

### 4.9. Nesfatin

Nesfatin-1, an appetite-suppressing adipokine primarily synthesized by subcutaneous WAT, is derived from a precursor protein called nucleobindin-2 (NUCB2). Both Nesfatin-1 and NUCB2 are potent peptides that reduce food intake and body weight. The *NUCB2* gene encodes a precursor peptide of 396 amino acids, including a 24-amino acid signal peptide. Prohormone/proprotein convertase (PC) 1/3 and PC2 enzymes cleave NUCB2 into three fragments, resulting in the production of Nesfatin-1 (amino acids 1–82), Nesfatin-2 (amino acids 85–163), and Nesfatin-3 (amino acids 166–396) [114].

Nesfatin has been found to enhance the expression of pre-proinsulin messenger RNA, stimulate insulin release in response to glucose, and suppress glucagon secretion, indicating its involvement in carbohydrate metabolism. Similar to other anti-inflammatory adipokines, Nesfatin inhibits MAPK signaling pathways and reduces levels of inflammatory cytokines. Moreover, it decreases the generation of ROS and enhances the activity of superoxide dismutase, thereby influencing the impact of oxidative stress [1]. Nevertheless, other studies have indicated mixed effects of Nesfatin. For example, Schalla et al. [115] reported that Nesfatin-1 administration significantly affects the cardiovascular system in preclinical animal models, particularly by increasing blood pressure. This effect is likely associated with activating the PI3K/AKT/mechanistic target of the rapamycin (mTOR) pathway and the phosphorylation of JAK2/STAT3. These signaling events lead to the proliferation, migration, and phenotypic transition of VSMCs, moving them from a state of contraction to a state of synthesis. This change is accompanied by increased mRNA and protein levels of MMP2 while decreasing the amounts of PPARγ [115].

In individuals diagnosed with essential hypertension, it has been observed that circulating levels of NUCB2/Nesfatin-1 are higher compared to normotensive individuals. These elevated levels are also positively correlated with systolic blood pressure. As a result, Nesfatin-1 has been proposed as a potential risk factor for hypertension associated with obesity, with an odds ratio of 1.5 [115,116].

### 4.10. Isthmin-1 (ISM1)

The *ISM1* gene, found on chromosome 20, encodes a protein of approximately 60 kDa. This protein consists of 499 amino acids and possesses three α-helices and two β-sheets in its structure [117]. ISM1 exhibits high expression in the isthmus organizer, a signaling center located at the midbrain and hindbrain (MHB) boundary. In zebrafish, the expression of isthmin was stimulated by Wnt/β-catenin overexpression, suggesting a role for ISM1 in embryonic development. Furthermore, Xiang et al. [118] demonstrated that ISM1 inhibits angiogenesis in vitro and in vivo by triggering endothelial cell apoptosis.

ISM1 is a recently discovered adipokine that inhibits hepatic lipid synthesis and enhances glucose uptake in adipose tissue. The signaling pathway of ISM1 relies on PI3K and involves a shared phosphorylation target with insulin signaling. Notably, its effects are not mediated through insulin receptors, suggesting a distinct mechanism of action [1]. In a research investigation led by Nguyen et al. [119], endogenous ISM1 was identified as a protective factor in a model of lipopolysaccharide (LPS)-induced acute lung injury (ALI). The research indicated that ISM1 possesses anti-inflammatory properties, potentially through inhibiting the NF-kB signaling pathway and the downregulation of inflammatory cytokines. These results imply that ISM1 modulates the inflammatory response and may have therapeutic potential in treating ALI and related conditions [119].

In a mouse model of obesity induced by a high-fat diet and nonalcoholic fatty liver, the administration of recombinant ISM1 via injection has shown promising results in reversing hepatic steatosis. This finding suggests that ISM1 may hold potential as a novel therapeutic approach for the clinical treatment of metabolic diseases, providing a new direction for further exploration in this field [120]. Findings from a study by Feng et al. [121] indicated a negative correlation between serum ISM1 levels and HDL among individuals diagnosed with T2DM [121]. Hence, further investigations are required to establish the connection between metabolic syndrome and ISM1.

### 4.11. Lipocalin-2 (LCN2)

Lipocalin-2 (LCN-2), also known as neutrophil gelatinase-associated lipocalin (NGAL), is a protein hormone comprising 198 amino acids. It functions as a circulatory protein that facilitates the transport of small hydrophobic molecules, including free fatty acids, steroids, prostaglandins, and hormones, to target organs. This transportation process occurs after LCN-2 binds to specific receptors such as megalin/glycoprotein and GP330 SLC22A17 or 24p3R LCN-2 [122].

Previous studies have linked endothelial dysfunction and hypertension in diet-induced obesity mouse models with the deamidated lipocalin-2 [123]. LCN-2 has been observed to be upregulated in conditions such as ischemia–reperfusion, coronary artery disease (CAD), and myocardial infarction. These findings indicate that LCN-2 has the potential to be a valuable biomarker for evaluating the prognosis and mortality risk of CVD [122]. In a Chinese cohort, Ni et al. [124] showed that increased levels of LCN2 in the bloodstream were linked to a higher occurrence of CAD and metabolic syndrome. These correlations suggest that the measurement of circulating levels of LCN2 may significantly predict the prevalence and prognosis of cardiometabolic diseases in this population [124]. Therefore, further investigation is required to shed light on the predictive value of this adipokine.

## 5. Brown Adipose Tissue (BAT) Adipokines (Batokines)

**Figure 4:**
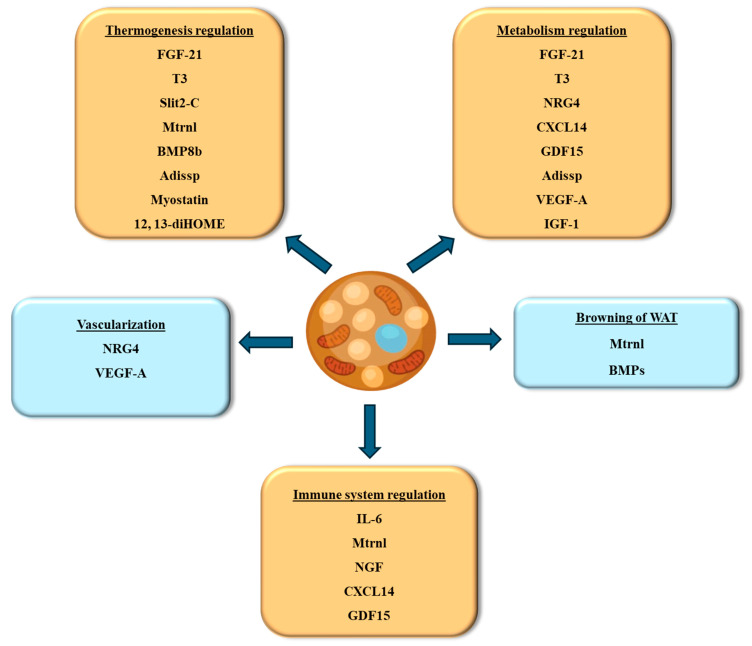
Batokines secreted from BAT and their functional roles. Abbreviations: IGF-1, insulin growth factor-1; CXCL14, chemokine (C-X-C motif) ligand 14; Mtrnl, meteorin-like; FGF-21, fibroblast browth factor 21; IL-6, interleukin-6; BMPs, bone morphogenetic proteins; NGF, nerve growth factor; 12,13-diHOME, 12,13-dihydroxy-9Z-octadecenoic acid; Adissp, adipose secreted signaling protein; T3, triiodothyronine; VEGF-A, vascular endothelial growth factor A; GDF15, growth differentiation factor-15; NRG4, neuregulin-4.

Batokines are signal molecules released by BAT. These molecules are critical in controlling metabolic processes, communicating with other tissues, and influencing overall energy balance. Batokines represent a vital aspect of metabolic regulation through their role in inter-organ communication and energy homeostasis. Their ability to enhance thermogenesis, improve insulin sensitivity, and influence lipid metabolism makes them promising targets for therapeutic interventions in obesity and metabolic disorders. Ongoing research into batokines will continue to uncover their full potential and pave the way for innovative treatments to improve metabolic health. The following section discusses the batokines identified to date and their role in preserving energy homeostasis (summarized in Figure 4).

### 5.1. Fibroblast Growth Factor-21 (FGF21)

FGF21, a peptide composed of 181 amino acids, was initially discovered in 2000. The gene responsible for encoding FGF21 is found on chromosome 19 in humans. It consists of four exons and encodes a precursor peptide known as pre-FGF21, which comprises 209 amino acids. Pre-FGF21 shares a high degree of similarity, with a 75% homology, to the murine FGF21 protein [125]. Research has demonstrated that FGF21 is not exclusively derived from the liver and WAT but also from BAT and beige adipose tissue [126]. FGF21 regulates various metabolic functions, including ketone body formation, fatty acid oxidation, and the physiological adaptation to starvation. Additionally, research has demonstrated that FGF21 directly triggers UCP1-mediated thermogenesis in brown and beige adipose tissue [127]. During cold exposure and beta-3-adrenergic stimulation, the transcription factor PPARα regulates the expression of FGF21. Under these conditions, FGF21 is upregulated in BAT, indicating its role in the adaptive response to cold and sympathetic nervous system activation [128].

The pleiotropic protein FGF21 transmits its signals via cell-surface complexes formed by fibroblast growth factor receptors and the transmembrane protein β-Klotho [129,130]. The expression of β-Klotho is primarily limited to metabolically active tissues such as the pancreas, liver, and adipose tissue. This restricted expression pattern allows FGF21 to target these metabolic tissues specifically. Due to this selective nature, FGF21 significantly impacts glucose and lipid metabolism, ultimately affecting body weight [130,131]. A study conducted by Hondares et al. [13] revealed that BAT serves as both a target and a significant source of FGF21. BAT produces FGF21 in response to thermogenic activation, a process regulated by a Cyclic adenosine 3,5-monophosphate (cAMP)-mediated pathway. The noradrenergic stimulation influences the gene transcription of FGF21, highlighting the role of this pathway in FGF21 production in BAT [13]. Furthermore, a clinical study conducted by Hanssen et al. [132] demonstrated the potential of FGF21 administration in improving metabolic consequences of obesity, such as dyslipidaemia and T2DM.

FGF21, a metabolic regulator that controls carbohydrate and lipid metabolism, has been demonstrated to enhance insulin sensitivity and promote glucose uptake. Additionally, it has been found to inhibit lipogenesis and promote the suppression of lipid oxidation. These effects of FGF21 contribute to regulating glucose and lipid homeostasis in the body [133]. In addition, FGF21 regulates critical cellular processes in macrophages, vascular endothelial cells, and vascular smooth muscle cells, protecting against the development of atherosclerosis and CAD [134]. FGF21 has also been shown to protect against myocardial infarction (MI) and post-MI ventricular arrhythmia by regulating inflammation, fibrosis, and the action potential duration of cardiac myocytes [134]. These effects contribute to the overall improvement in cardiac function and the reduction in adverse events following MI. Clinical studies have revealed direct correlations between reduced serum FGF21 levels and the development of various CVDs, such as myocardial ischemia, CAD, cardiac hypertrophy, atherosclerosis, and diabetic cardiomyopathy, supporting the protective function of endogenous FGF21 against CVDs. FGF21 has been shown to facilitate multiorgan communication between the liver, adipose tissue, and blood vessels, which in turn leads to increased lipid oxidation and suppressed lipid accumulation. Ultimately, this contributes to the prevention of atherosclerosis and other cardiometabolic diseases [133].

### 5.2. Triiodothyronine (T3)

Triiodothyronine (T3) is a crucial hormone in various physiological functions, such as heart rate regulation, thermogenesis, metabolism, and development. In the late 1980s, T3 was initially identified as a hormone released by BAT [135]. Identifying T3 as a hormone secreted by BAT was supported by evidence demonstrating that brown adipocytes regulate the activity of type 2 iodothyronine deiodinase (DIO2) in response to norepinephrine stimulation. This enzymatic activity facilitates the conversion of thyroxine (T4) to T3 within brown adipocytes [136]. BAT has a notable function in regulating systemic levels of T3. Previous studies suggest that BAT generates and disseminates T3 in the bloodstream [135]. T3 stimulates thermogenesis in BAT by promoting the expression of UCP1 in response to cold temperatures [137]. In fact, the presence of T3 is essential for the complete activation of thermogenesis during cold exposure. The activation of DIO2 takes place through beta-adrenergic signaling in brown or white adipocytes, and the T3 produced locally acts on the T3 receptor beta isoform (TRβ1), which is crucial for beginning thermogenesis [136,137,138,139].

A study conducted recently by Liu et al. [140] has demonstrated that prolonged administration of T3 in male mice leads to enhanced recruitment of thermogenic capacity in the interscapular BAT. This effect is achieved by promoting adipocyte progenitor cell proliferation mediated by the thyroid hormone receptor α (THRA). The increased proliferation of these cells results in hyperplasia, contributing to the expansion of the BAT and facilitating adaptive thermogenesis. The mechanism by which T3 generated in brown or beige adipocytes may function in an endocrine capacity remains incompletely elucidated. Consequently, additional investigation is necessary to ascertain whether T3 produced in these adipocytes can influence distant tissues through an endocrine signaling route [141].

### 5.3. Interleukin-6 (IL-6)

The *IL-6* gene, found on chromosome 7 at the 7p21-p14 location, comprises five exons and spans a length of 5 kilobases. Within the *IL-6* gene promoter, multiple regulatory sites facilitate the activation of gene expression through various mechanisms, such as glucocorticosteroids and cAMP [142]. Different tissues produce IL-6 in response to tissue injuries and inflammation. IL-6 plays a vital role in fever and the acute phase response. The source of IL-6 production significantly impacts the outcomes. For example, IL-6 produced by fat cells encourages the infiltration of macrophages into adipose tissue, whereas IL-6 produced by myeloid cells and muscles hinders this process. These opposing effects are due to a change in IL-6 downstream signaling. While myeloid cells utilize a conventional signaling mode, adipocytes and muscles engage in a noncanonical trans-signaling mode. This alteration is accompanied by increased disintegrin and metalloproteinase domain-containing protein 10/17 (ADAM10/17) expression, which enables trans-signaling via the soluble IL-6 receptor α [143].

In cardiometabolic disorders such as atherosclerosis and diabetes, the progression of complications that result from pro-inflammatory and autoimmune mechanisms is significantly influenced by the presence of IL-6. Individuals diagnosed with congestive heart failure (CHF) have shown increased levels of IL-6 in the circulation and within the heart tissue, even when other cytokines, such as TNF-α, were within normal range [144]. Similarly, patients with left-sided heart failure exhibited elevated concentrations of circulating IL-6, associated with the severity of left ventricular dysfunction and the activation levels of the sympathetic and renin-angiotensin systems. Higher IL-6 levels were also correlated with a lower cardiac functional class, reduced ejection fraction, and an unfavorable prognosis [144]. IL-6 is a crucial inflammatory factor associated with the advancement and worsening of atherosclerosis. Increased concentrations of IL-6 in the blood, in conjunction with various other cytokines, have been connected to adverse clinical consequences in individuals hospitalized for unstable angina. The concentration of IL-6 is not only indicative of disease severity but also serves as a highly accurate predictor of disease outcomes [145].

Experimental studies have shown the stimulation of β3-adrenergic receptors to enhance the expression and release of IL-6 in mouse brown adipocytes [146]; yet, the exact mechanism of IL-6 stimulation remains unclear and requires further investigation. In support of the significant role of BAT-produced IL-6, Stanford et al. [147] observed the occurrence of liver inflammation and widespread insulin resistance when BAT lacking IL-6 was transplanted into the abdominal cavity of mice. Conversely, transplantation of IL-6-producing BAT increased insulin-stimulated glucose uptake in various tissues, including endogenous BAT, WAT, and heart muscle. These findings underscore the critical function of IL-6 derived from BAT in glucose regulation and insulin sensitivity [147].

### 5.4. Meteorin-like (Mtrnl)

Metrnl is an adipokine whose expression in BAT and skeletal muscles is induced by cold exposure and physical activity [148]. Elevated levels of circulating Metrnl have been shown to cause an energy deficit by enhancing thermogenesis and overall energy expenditure in WAT. Interestingly, Metrnl may not directly interact with adipocytes to regulate the thermogenic gene program. Instead, it stimulates various immune cell subtypes to infiltrate the adipose tissue microenvironment and elicit their prothermogenic effects [15]. Metrnl is crucial in rodent and human metabolic adaptations to cold temperatures. It induces a type 2 immune response characterized by the involvement of eosinophils and the release of IL-4. This immune response leads to the alternative activation of adipose tissue macrophages. Additionally, Metrnl suppresses type 1 inflammatory cytokines, further contributing to regulating immune and metabolic processes in response to cold exposure [149]. The alternatively activated macrophages upregulate the thermogenic genes in UCP-1-expressing beige adipocytes. Consequently, Metrnl is a regulator linking the host’s adaptive reactions to managing tissue inflammation and energy balance [15].

The molecular mechanism by which Metrnl induces whole-body energy expenditure involves two main pathways. First, Metrnl activates the STAT6 pathway, transforming white adipose cells into beige or brown-like adipocytes, which are more metabolically active. Second, Metrnl activates the PPARγ-mediated pathway, which regulates adipocyte differentiation and lipid metabolism. This dual activation of STAT6 and PPARγ pathways contributes to the overall increase in energy expenditure and metabolic effects induced by Metrnl [148]. Research conducted on mouse models of obesity and diabetes has demonstrated that increased circulating levels of Metrnl result in the browning of white fat depots, associated with increased energy expenditure and improved glucose tolerance at the whole-body level [15].

Previous studies have shown that Metrnl expression is reduced in the cardiac tissues of spontaneously hypertensive rats, and increasing its expression alleviates hypertension and pathological cardiac hypertrophy [150]. This beneficial effect is thought to be achieved through activating the BReast CAncer gene 1 (BRCA2)/Akt/mTOR signaling pathway and inhibiting excessive autophagy [150]. Low plasma levels of Metrnl are associated with markers of cardiac damage, such as troponin, markers of renal damage, including creatinine and urea, and markers of hepatic damage, such as alanine aminotransferase and albumin. Furthermore, lower plasma levels of Metrnl are linked to the heart failure marker, N-terminal pro-B-type natriuretic peptide (NT-proBNP), and the echocardiographic parameters of heart failure, such as intraventricular septum and left ventricular posterior wall thickness. These connections imply that Metrnl could serve as a promising indicator for evaluating cardiac function and injury in CVD [151].

Exercise and increased physical activity were found to enhance the production of Metrnl in individuals diagnosed with CAD. This increase in Metrnl levels subsequently led to improvements in atherosclerosis by reversing endothelial dysfunction and inflammation. The effect of induced Metrnl levels on ameliorating vascular inflammation among CAD patients was mediated by decreasing the NLR family pyrin domain containing 3 (NLRP3) inflammasome activity. These findings suggest that exercise-induced elevation of Metrnl can potentially mitigate inflammatory processes and promote vascular health in individuals with CAD [152].

### 5.5. Bone Morphogenetic Proteins

Bone morphogenetic proteins (BMPs) are part of the TGFβ superfamily of signaling proteins. As multifunctional regulators, they play essential roles in developing and maintaining tissue homeostasis [153]. Certain BMP group members, such as BMP2, BMP4, BMP7, and BMP8B, have been recognized for their significant contributions to regulating different stages of adipose tissue development in various locations [154]. BMPs are crucial in controlling the molecular mechanisms responsible for the differentiation of beige and BAT [155]. The signaling of BMPs involves the activation of SMAD transcription factors through the serine-threonine kinase receptors BMPR1 and BMPR2, which mediate the downstream effects of BMP signaling [153,156]. The modulation of blood levels of different BMPs and the expression of genetic forms of BMPR1A and BMPR2 in adipocytes have been linked to obesity development and fat distribution. BMP-7, mainly generated by stromal vascular cells in BAT, contributes to brown adipogenesis by inducing brown adipocyte differentiation. This protein is crucial for the formation of classic BAT depots.

In a mouse model of atrial fibrillation (AF), BMP-7 has been shown to protect cardiac functions. BMP-7 achieves this by regulating TGF-β1/Smad3 signaling through interacting with Smad1/5. This interaction allows BMP-7 to counteract fibrosis in myocardial fibroblasts that occur due to atrial fibrillation. By antagonizing fibrosis, BMP-7 helps to preserve cardiac function in AF [157]. Administering BMP-7 from an external source reduces inflammation and cardiac remodeling, enhancing left ventricular (LV) function in diabetic mice [158]. BMP-7 demonstrates its ability to improve cardiac function across various heart diseases. Additionally, BMP-7 promotes the polarization of pro-inflammatory M1 macrophages in the heart tissues into anti-inflammatory M2 macrophages in different cardiac conditions [159].

Another significant BMP, BMP8b, is mainly produced by mature brown adipocytes. The expression of BMP8b increases when exposed to cold temperatures or a diet high in fat [160]. BMP8b amplifies BAT’s response to β3-adrenergic stimulation and boosts the activation of the p38 MAPK pathway [160]. BMP8b also enhances signaling within BAT by activating SMAD1, SMAD5, and SMAD8. Interestingly, the presence of BMP8b in BAT is notably more elevated in female mice than male mice. This phenomenon could be attributed to the ability of estrogen to induce BMP8b expression [161], which in turn could explain the observed gender-specific variances in BAT function in mice. Apart from its influence within BAT, BMP8b functions in the hypothalamus, heightening BAT thermogenesis stimulation through the sympathetic nervous system. Overall, BMP8b serves a dual purpose by enacting local impacts in BAT and central effects in the hypothalamus to enhance BAT activation and thermogenesis through the sympathetic nervous system [160].

### 5.6. Neuregulin-4 (Nrg4)

Neuregulin-4 (Nrg4) is a critical component of the epidermal growth factor (EGF) protein family, featuring a bioactive EGF-like domain that connects it to the tyrosine kinase ErbB receptors [162]. Although Nrg4 is produced and released by all types of adipose tissues, it shows a greater abundance in BAT and WAT when subjected to cold conditions [163]. Low levels of *Nrg4* gene expression have been observed in the liver despite being a critical insulin-sensitive tissue. Also, there is no clear evidence of Nrg4 expression in tissues such as the heart or skeletal muscle [163,164]. Nrg4 has been proposed as an adipokine with endocrine effects, meaning it can act as a signaling molecule in various tissues throughout the body. In conditions associated with insulin resistance, such as obesity and T2DM, the expression of Nrg4 in adipocytes is reduced [164]. Multiple research studies have illustrated the involvement of Nrg4 in preserving vascular performance in both BAT and WAT. Genetic loss of *Nrg4* has been associated with impaired BAT vascularization, obesity, and metabolic dysfunction in chow diet-fed mice [165], supporting the critical role of Nrg4 in regulating adipose tissue vascular and metabolic functions [165].

Elevated levels of Nrg4 have various positive effects on regulating metabolic homeostasis through paracrine and endocrine pathways. These effects include promoting hepatic lipogenesis, enhancing fuel oxidation, supporting innervation, and stimulating angiogenesis. Additionally, in rodents, there is an inverse relationship between Nrg4 levels and the occurrence of T2DM and NAFLD [164]. Consistent with findings in rodents, decreased Nrg4 circulating levels were observed in individuals with T2DM, gestational diabetes, metabolic syndrome, and CAD, suggesting a protective role of Nrg4 against the development or progression of these metabolic and cardiovascular conditions [166,167,168].

### 5.7. Nerve Growth Factor (NGF)

Brown adipocytes synthesize and release nerve growth factor (NGF). Interestingly, the production of NGF is negatively correlated with sympathetic activity, irrespective of whether in physiological or pathophysiological conditions. NGF production was reported to be higher in genetic animal models of obesity [169] and in obese women with metabolic syndrome, which was linked to low-grade systemic inflammation [170,171]. Exposure to cold temperatures in mice increased NGF synthesis, specifically in BAT. This increase was associated with enhanced neurite outgrowth, promoting nerve fiber growth and branching [172]. When 3T3-L1 adipocytes were exposed to an inflammatory cytokine-like TNF-α, there was a notable rise in the expression and secretion of NGF [173]. NGF functions as both an inflammatory mediator and a neurotrophic factor in adipose tissue, allowing it to regulate inflammatory responses and promote the proliferation and function of nerve cells in adipose tissue. [174,175]. Changes in the levels or distribution of NGF and its receptors are believed to play a role in developing various vascular and heart diseases, such as myocardial infarction, hypertension, cardiac hypertrophy, heart failure, atherosclerosis, and acute coronary syndromes (ACS) [176]. However, additional investigation is required to gain a deeper understanding of the function of NGF and its contribution to the development of cardiometabolic diseases.

### 5.8. S100B Protein

The calcium and zinc-binding protein, S100B, is expressed in brown/beige and WAT [177]. The expression of S100B is not limited to adipocytes alone. It is also found in various other cell types, including nervous tissue, chondrocytes, melanocytes, Leydig cells, myoblasts, skeletal muscle cells, dendritic cells, and specific populations of lymphocytes [178,179,180]. In general terms, the protein S100B, like other calcium-binding proteins in the S100 family, regulates various cell activities. It accomplishes this by engaging with multiple molecules in diverse cell types [181], such as calsyntenin 3β (CLSTN3β), a mammalian protein of the endoplasmic reticulum (ER) that has a crucial function in the sympathetic innervation of both WAT and BAT [182]. S100B acts as a downstream mediator of the signaling pathway triggered by CLSTN3β in reaction to cold exposure [182]. Ca^2+^/Zn^2+^-dependent S100B protein also engages with intracellular proteins, overseeing post-translational alterations like phosphorylation, transcriptional functions, enzymatic operations, and the arrangement and oxidation of various cytoskeletal elements [183,184,185].

The *S100B* gene is regulated by the transcription factor PRDM16, which activates the thermogenic program in brown adipocytes. Several identified or suspected intracellular targets of S100B, such as p53, ATAD3A (ATPase family AAA domain-containing 3A), CYP2E1 (Cytochrome P450 Family 2 Subfamily E Member 1), and AHNAK (AHNAK Nucleoprotein), have roles related to the physiology of adipose tissues. In addition to its intracellular roles, S100B is secreted by adipocytes in response to stimulation of β-adrenergic receptors. In this extracellular capacity, S100B acts as a neurotrophic factor involved in the sympathetic innervation of thermogenic fat [182]. S100B levels in the blood were elevated in obese individuals and positively correlated with insulin resistance, serum triglyceride levels, and abdominal adiposity [186]. In addition, S100B could predict more accurately the outcome in the early phase of cancer compared with other current biomarkers [187,188].

### 5.9. C-X-C Motif Chemokine Ligand-14 (CXCL14)

CXCL14 belongs to the CXC cytokine family and is recognized as a potent chemoattractant. Its role involves facilitating the migration of immune cells, including monocytes, dendritic cells, and natural killer (NK) cells, to specific areas of inflammation. This chemotactic effect enables these immune cells to localize and accumulate at sites of inflammation in response to inflammatory signals [189]. CXCL14, in addition to its role as a chemoattractant, is also identified as a batokine produced by metabolically active BAT. Previous studies have reported that CXCL14 can enhance glucose metabolism in rodent models with glucose intolerance and insulin resistance [189]. Its production by BAT suggests a potential role in regulating glucose homeostasis and improving metabolic health.

CXCL14, secreted by brown adipocytes, plays a role in recruiting or polarizing M2-like anti-inflammatory macrophages. In rodent models, the absence of CXCL14 leads to disrupted glucose homeostasis and altered activity of BAT. Moreover, in a mouse model of diet-induced obesity, CXCL14 promotes the conversion of WAT to a more metabolically active state (browning) and improves glucose and insulin regulation. This effect is achieved by activating type 2 immune cells in adipose tissue [189]. Another study on individuals with T2DM reported that circulating levels of CXCL14 could potentially serve as a valuable predictor of hepatic steatosis [190]. CXCL14 levels exhibit significant differences between individuals diagnosed with and without CAD, and these levels were significantly linked with myocardial function. Moreover, in individuals with symptomatic heart conditions, reduced levels of circulating CXCL14 are connected with unfavorable results [191]. Therefore, CXCL14 is a batokine that regulates the immune function and several aspects of the cardiometabolic function.

### 5.10. Growth and Differentiation Factor-15 (GDF15)

GDF15, a pleiotropic batokine, exhibits various physiological and pathological effects, including modulating inflammatory responses, regulating metabolism, and contributing to tumorigenesis [192]. In mice, the expression of GDF15 in brown adipocytes was found to be stimulated in response to thermogenic stimuli and high-fat feeding [193]. GDF15 expression and release from brown adipocytes are induced by norepinephrine and cAMP by protein kinase A-mediated processes. This noradrenergic regulation of GDF15 relies on the active FGF21 pathway in brown adipocytes. GDF15 acts on adipose-associated macrophages in a paracrine manner, alleviating pro-inflammatory pathways in the local adipose tissue environment [194]. In a UCP1-specific GDF15 transgenic mouse model, Jena et al. [193] demonstrated that GDF15 derived from brown adipocytes is essential for enhancing insulin sensitivity and regulating weight gain caused by a high-calorie diet. GDF15 was shown to control the expression of the *IL6* gene while inducing the differentiation of human embryonic stem cells (hESCs) into brown adipocytes [192].

In healthy conditions, GDF-15 has been found to have various beneficial effects, such as reducing appetite, decreasing inflammation, and increasing insulin sensitivity. In certain conditions like obesity, diabetes, and chronic inflammation, GDF-15 is upregulated. It has been suggested that receptors for GDF-15 may develop resistance, which could explain the significant increase in circulating GDF-15 levels observed during disease states [195]. Observational studies conducted on healthy individuals have revealed an association between elevated levels of GDF-15 and a higher risk of cardiovascular events over time. Additionally, in individuals with conditions such as CAD and heart failure, GDF-15 has been correlated with an increased risk of overall mortality and adverse events. These findings suggest that GDF-15 may serve as a valuable prognostic marker in patients with high cardiometabolic risk [196].

### 5.11. Myostatin

Myostatin, alternatively referred to as growth and differentiation factor-8 (GDF8), is a batokine that regulates skeletal muscle function [197]. Myostatin secretion is directly associated with the degree of thermogenic stimuli and WAT browning [198,199]. It demonstrates dual functionality in adipogenesis, as its effects can either promote or inhibit the process, contingent upon the physiological conditions [200]. Myostatin is recognized as a negative regulator of skeletal muscle growth [201]. When BAT is deactivated, such as through exposure to a thermoneutral temperature, myostatin levels tend to increase, decreasing exercise capacity in skeletal muscle. Conversely, the activation of BAT through experimental means leads to reduced levels of myostatin and improved exercise performance. Surgical removal of BAT has been shown to attenuate the inhibitory effects of myostatin on muscle tissues [197]. Furthermore, studies have shown that the absence of myostatin leads to myocardial hypertrophy, while its overexpression diminishes heart mass. These data imply that myostatin limits hyperplastic growth, cardiomyocyte proliferation, and the rate of protein synthesis in the developing heart [202].

### 5.12. Adipose Secreted Signaling Protein (Adissp)

The adipose secreted signaling protein (Adissp), also known as the UPF0687 protein or human C20orf27 homolog, plays a crucial role in regulating thermogenesis in WAT and maintaining glucose balance in the body. Adissp is primarily expressed in adipose tissues; its mRNA levels are abundant in BAT, and its secretion is triggered by the activation of β3-adrenergic receptors [203]. In preclinical studies, Adissp has been found to enhance thermogenesis in WAT, improve glucose regulation, and provide protection against obesity. When Adissp is specifically knocked out in adipose tissue, mice show impaired browning of WAT and increased susceptibility to hyperglycemia and obesity induced by a high-fat diet. Mechanistically, Adissp activates protein kinase A through a pathway independent of β-adrenergic signaling by binding to a potential receptor on adipocyte surfaces. In general, Adissp is primarily produced by BAT and serves as a substantial upstream signaling component in the thermogenesis process [203].

### 5.13. Slit2-C

Slit, an extracellular matrix protein, comprises three homologs: Slit1, Slit2, and Slit3. It has been discovered that beige adipocytes can release Slit2 in a cleaved form known as Slit2-C in response to thermogenic stimulation. The secreted Slit2-C can activate the PKA signaling cascade, enhancing adipose thermogenesis. This effect has been demonstrated to improve energy expenditure and glucose regulation in mice [204]. In their clinical investigations, Kang et al. [205] demonstrated lower circulating levels of Slit2 in diabetic patients. Slit2 also suppresses inflammatory reactions and preserves myofilaments’ contractile properties. This mechanism contributes, at least partially, to the protection against structural and functional damage in cases of ischemia–reperfusion injury. By inhibiting inflammation and maintaining the contractile properties of myofilaments, Slit2 helps prevent the detrimental effects associated with the interruption and subsequent restoration of blood flow during ischemia–reperfusion injury [206]. In support of these findings, recombinant Slit2 has shown potential in alleviating inflammation, myocardial fibrosis, and oxidative stress in CAD [207]. However, more clinical and mechanistic research is necessary to thoroughly comprehend this adipokine’s function.

### 5.14. Vascular Endothelial Growth Factor A (VEGF-A)

Proper control of the vasculature is crucial for maintaining the functions of both BAT and WAT. It is conceivable that vascularization, or the formation of blood vessels in adipose tissue, relies, at least in part, on the signaling of a protein called VEGF-A [208]. Experimental findings from previous studies have verified the importance of the VEGF-A-vascularization axis in thermogenesis. When VEGF-A is overexpressed in adipocytes, it induces vascularization and stimulates the expression of UCP1 in both WAT and BAT. This enhancement of vascularization and UCP1 expression leads to an overall improvement in thermogenesis. Conversely, when VEGF-A is depleted in the adipocytes of normal mice, thermogenesis is inhibited, indicating that VEGF-A-induced vascularization plays a critical role in activating the thermogenic process [208,209]. VEGF-A has surfaced as a hopeful target for tackling insulin resistance linked with obesity. Injecting VEGF-A directly into the adipose tissue of obese mice has been proven to reinstate vascularity, ameliorate compromised insulin sensitivity, and boost overall glucose metabolism [209]. VEGF-A is crucial in cardiac morphogenesis, cardiac contractility, and myocardial wound healing. Nevertheless, elevated serum levels of VEGF-A were observed in various CVDs, frequently correlating with unfavorable prognoses and increased disease severity [210]. These results may suggest that the function of VEGF-A is contingent upon its source of production and its target tissue. Additionally, it has a dual impact on cardiometabolic function, necessitating strict regulation of its levels.

### 5.15. Insulin Growth Factor-1 (IGF-1)

IGF-1, a peptide known for its cell growth properties, is vital in growth, maturation, and tissue specialization. It influences cells through endocrine, paracrine, and autocrine pathways. The importance of IGF-1 as a batokine was highlighted in a study involving BAT transplantation in mice with type 1 diabetes, which showed a notable and swift rise in IGF-1 expression within the BAT, coupled with increased levels of circulating IGF-1. Furthermore, BAT transplantation and IGF-1 induction reduced pro-inflammatory responses and improved glucose homeostasis [211]. Similarly, exposure to cold temperatures elevated the expression of IGF-1 in rats. This upregulation of IGF-1 was found to induce BAT cells’ hyperplasia and cell proliferation [212]. Additionally, IGF-1 has been demonstrated to reduce insulin levels, enhance insulin sensitivity, and facilitate glucose metabolism [212]. In humans, IGF-1 has been found to stimulate contractility and tissue remodeling in the heart, leading to improved heart function following a myocardial infarction [213]. Prior studies suggest that IGF-1 plays a protective role against the progression of atherosclerosis and that decreased levels of circulating IGF-1 have been linked to a higher likelihood of developing CVD [214].

Clinical studies of adipokines have provided significant insights into their roles in health and disease, particularly in relation to obesity, metabolic syndrome, cardiovascular diseases, and other chronic conditions. Different adipokines, such as adiponectin, leptin, resistin, chemerin, apelin, visfatin, and omentin, exert effects on insulin sensitivity, inflammation, vascular function, energy metabolism, bone health, and others. Comprehending the diagnostic consequences of these adipokines can facilitate the development of specific treatments for controlling obesity, T2DM, metabolic syndrome, and cardiovascular disorders. Ongoing research continues to explore the complex roles of adipokines in different health conditions and their potential as therapeutic targets. Representative clinical investigations that addressed adipokine dysregulations in various populations and under various health conditions are presented in Table 1.

## 6. Role of Adipokines in the Adipose-Organ Crosstalk

**Figure 5:**
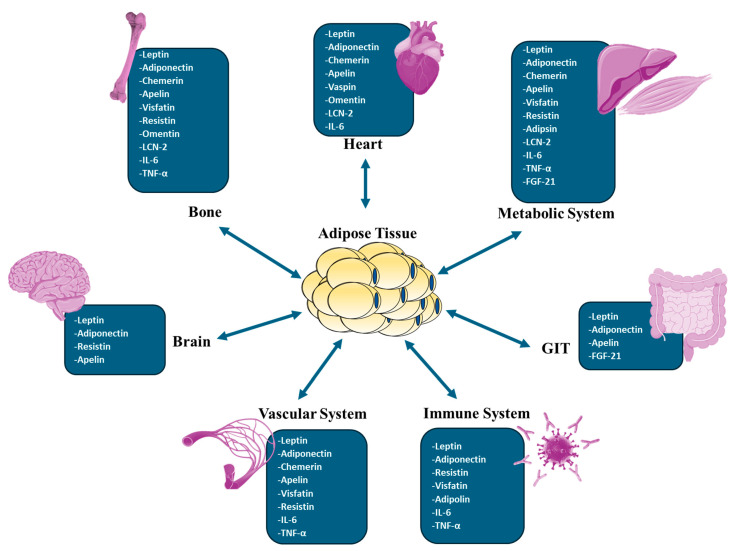
The most prominent adipokines in adipose tissue-organ crosstalk. FGF21, fibroblast growth factor 21; IL-6, interleukin-6; TNF-α, tumor necrosis factor alpha; LCN-2, lipocalin 2.

Adipose-organ crosstalk refers to the intricate communication between adipose tissue and various organs in the body. This interaction is crucial for maintaining metabolic homeostasis, regulating energy balance, and influencing overall health. Adipokines are essential mediators of this crosstalk between adipose tissue and other organs, influencing various physiological and pathological processes. This section highlights some prominent examples of the interaction between adipose tissue and other organs (Figure 5).

### 6.1. Adipose Tissue–Brain Axis

The brain plays a vital role in regulating the body’s overall metabolic state, ensuring a balanced and stable energy metabolism. It achieves this by coordinating various networks within the body, including specific brain regions and peripheral organs like the adipose tissue, the gastrointestinal tract, and the pancreas. These networks function harmoniously to regulate short-term and long-term processes that maintain the body’s energy balance [218]. Signals from the periphery, transmitted through the bloodstream and afferent neurons, interact with specific areas in the brain. These interactions either stimulate or inhibit specific neurons, contributing to maintaining energy homeostasis through both neuronal and endocrine responses [219]. The arcuate nucleus (ARC) and ventromedial hypothalamus (VMH) are key brain regions that are essential for regulating metabolism. These regions express receptors for adipokines, such as leptin [220]. The crosstalk and synchronization of endocrine signals, brainstem, and hypothalamic centers enable the body’s natural regulation of satiety, fat accumulation, and the size and frequency of meals [218,219].

The hypothalamus plays a fundamental role in regulating food intake. More specifically, specific nuclei within the hypothalamus, such as the ARC, paraventricular nucleus (PVN), VMH, dorsomedial hypothalamus (DMH), and lateral hypothalamic area (LHA), form an interconnected network of neurons. These nuclei are connected to both the brainstem and higher brain centers, contributing to the overall regulation of food intake [221]. These brain centers detect and react to fluctuations in the body’s energy levels and the quantity of food consumed [222]. By perceiving these factors, the hypothalamic nuclei and brain centers work together to modify the pattern of expression of specific neuropeptides, ultimately regulating food intake and energy expenditure and ensuring the maintenance of energy homeostasis [223,224].

The arcuate nucleus (ARC) is a hypothalamic nucleus containing hormone receptors and nutrient sensors. It expresses receptors for various hormones, including insulin, leptin, and ghrelin. Additionally, the ARC is situated immediately above the median eminence, a circumventricular organ characterized by a semipermeable blood–brain barrier (BBB). This unique characteristic allows peptides and proteins to enter the ARC from the bloodstream [218,223,225]. The hypothalamic ARC serves as the primary detector of nutrient signals and the circulating peripheral hormones originating from the adipose tissue and the gut [218,224,226]. It comprises two distinct neuronal populations that exert contrasting impacts on food consumption. The first population comprises NPY/AgRP neurons, which synthesize and release orexigenic neuropeptides. These neuropeptides include neuropeptide Y (NPY) and agouti-related peptide (AgRP), which are known to boost appetite [218,222,227]. The second group of neurons in the hypothalamic ARC consists of POMC and cocaine- and amphetamine-regulated transcript (CART) neurons. These neurons produce anorexigenic neuropeptides, specifically POMC and CART. These neuropeptides inhibit food intake and suppress appetite [228]. Leptin, insulin, and ghrelin mediate peripheral metabolic signals that primarily exert their actions on the first-order neurons, including NPY/AgRP and POMC/Cocaine and amphetamine-regulated transcript (CART) neurons, within the hypothalamic ARC. These signals substantially regulate the activity and function of these neurons, thereby influencing food intake and energy balance [223,224,226,229].

In the crosstalk between adipose tissue and the CNS, the most extensively studied adipokines in terms of their functions and specific signaling pathways are leptin, adiponectin, resistin, and apelin [230,231,232,233,234]. Leptin is essential for controlling food intake and energy expenditure by interacting with specific brain centers. It acts as a natural antagonist to the hunger hormone ghrelin, secreted by the stomach, small intestine, and pancreas [235]. Leptin resistance, which refers to the brain areas such as the hypothalamus and hindbrain being unable to respond to plasma leptin levels, could potentially contribute to the onset of obesity. In a lean and healthy state, leptin signals from adipose tissue effectively communicate with the CNS regarding long-term adipose tissue fat storage levels. However, leptin resistance disrupts this communication, misinterpreting adipose tissue fat storage levels. This disruption results in hyperphagia and hyperglycemia, key metabolic syndrome symptoms [220].

The brain was found to express Adiponectin receptors, including Adipo-R1, Adipo-R2, and T-cadherin. Adipo-R1 is predominantly expressed in the hypothalamus, brainstem, and pituitary gland, while Adipo-R2 is primarily expressed in the cortex. The cerebral cortex, basal ganglia, amygdala, and hippocampus exhibited extensive T-cadherin expression [236]. Through these receptors, adiponectin influences a diverse array of metabolic and vascular processes, such as glucose and lipid metabolism, insulin sensitivity, anti-atherogenic activity, and vascular function, as well as regulating body weight. Various signaling pathways mediate these effects, including AMPK, p38-MAPK, JNK, PPAR-α, and NF-kB [237]. Furthermore, adiponectin has been reported to prevent the activation of pro-inflammatory pathways by inhibiting the secretion of IL-6 and TNFα from endothelial cells of the blood–brain barrier [238]. Previous research has also shown that adiponectin has neuroprotective effects through the AMPK pathway and that adiponectin knock-out mice have more significant brain damage following an ischemic stroke than wild-type mice. This neuroprotective effect is obtained through an eNOS-dependent mechanism [239].

Resistin is another adipokine crucial in communication between adipose tissue and the CNS. It specifically affects the activity of the sympathetic nervous system, but in an organ-specific manner. For example, resistin has been demonstrated to enhance sympathetic activity in the skeletal muscle vasculature and kidneys, hence contributing to the elevated blood pressure and cardiovascular problems observed in individuals with obesity and diabetes. Conversely, resistin inhibits sympathetic activity to BAT, leading to a decrease in thermogenesis and a reduction in energy expenditure [240,241].

Limited research has been conducted on the role of other adipokines, including retinol-binding protein-4 (RBP4), omentin, and visfatin, in the communication between adipose tissue and the brain. However, studies involving RBP4-deficient mice have provided some insights. Behavioral observations of these mice have indicated decreased locomotor activity and increased anxiety-like behavior. Histological analysis has revealed neuronal loss and gliosis in the cortex and hippocampus, as well as a reduction in the proliferation of neuroblasts in the subventricular zone. These findings suggest potential associations between RBP4 deficiency and neurobehavioral alterations. Nevertheless, further investigation is required to gain a comprehensive understanding of the involvement of these adipokines in adipose tissue-brain crosstalk [242]. Omentin, on the other hand, has been found to exert orexigenic effects, stimulating appetite. It has been shown to decrease the gene expression of CART and corticotropin-releasing hormone (CRH) in the hypothalamus. Additionally, omentin can increase the synthesis and release of norepinephrine (NE) from the hypothalamus [243]. In contrast, visfatin has been found to induce anorexia and promote homeostatic feeding behavior. It reduces food consumption and body weight in mice by activating POMC neurons and microglia [244].

In summary, adipokines exhibit both pro-inflammatory and anti-inflammatory activities, and maintaining a balanced production of these molecules is crucial for homeostasis. Obesity-induced adipose tissue dysfunction disrupts the balanced production of adipokines, resulting in local and systemic effects on inflammatory cells. This imbalance of adipokines contributes to a chronic, low-grade inflammatory state that plays a significant role in the development of metabolic and CVD.

### 6.2. Adipose-Vascular Crosstalk

Adipose tissue comprises a heterogeneous cell population of adipocytes, fibroblasts, stem cells, immune cells, and endothelial cells. The intercellular communication and crosstalk between these different cell types play a crucial role in regulating adipose tissue function [245]. Crosstalk between adipocytes and blood vessels is essential for maintaining metabolic balance.

Adipocytes release extracellular vesicles (EVs) that facilitate cell-to-cell communication, influencing the function of distant organs and neighboring cells [246]. EVs encompass all the essential components within a cell, including nucleic acids, lipids, and various proteins originating from the nucleus, cytosol, and cell membrane [247,248]. These EVs are critical in normal physiological processes, cellular communication, and cellular waste removal [249]. Our research team has reported that EVs produced by dysfunctional adipose tissue in obese individuals exhibit disrupted metabolic profiles characterized by excess ceramides, glycosphingolipids, and inflammatory adipokines. These dysregulated metabolites adversely impact vascular function. In particular, we illustrated the role of adipose EVs obtained from obese subjects in the induction of endothelial cell caveolar fission and the disruption of endothelial nitric oxide signaling, endothelial cell permeability, and endothelial response to shear stress. Additionally, we have reported that the flow-induced dilation and nitric oxide production of lean, healthy arterioles are impaired when incubated with adipose EVs obtained from obese individuals [250].

In addition to EV cargo, soluble adipokines impact the function of blood vessels in paracrine and endocrine fashions. For instance, adiponectin is primarily expressed in perivascular adipocytes, while its receptors, AdipoR1 and AdipoR2, are found throughout the blood vessels. Within the vascular endothelium, adiponectin operates via AdipoR1 and AdipoR2 receptors to enhance nitric oxide generation through AMPK (adenosine monophosphate-activated protein kinase), stimulating eNOS signaling and resulting in vasodilation [251]. Besides its interaction with AdipoR1 and AdipoR2 receptors, adiponectin can also attach to endothelial cells via the T-cadherin receptor, which increases EV production by endothelial cells [252]. This process helps endothelial cells eliminate waste products and maintain cellular homeostasis [253]. Previous studies have shown that hypertension induced by angiotensin II (Ang II) leads to a significant decrease in adiponectin levels and the expression of AdipoR1 and AdipoR2 in both perivascular adipocytes and vascular cells. On the other hand, adiponectin exhibits a protective effect by inhibiting Ang II-induced p38 phosphorylation and subsequent proliferation and migration of VSMCs. In support of the vascular protective action of adiponectin, the synthetic adiponectin receptor agonist AdipoRon was found to mitigate Ang II-induced vascular hypertrophy and fibrosis [254].

Some adipokines have dual effects on blood vessels to maintain vascular tone in various conditions. Leptin, for instance, causes vasoconstriction by activating the sympathetic nervous system while also causing vasodilation by promoting nitric oxide generation [255]. However, the net effects of leptin typically involve vasodilation, which leads to decreased blood pressure when infused in vivo [256]. Similar dual effects are induced by TNF-α, which is produced by perivascular adipose tissue (PVAT). TNF-α is a potent regulator of vascular tone, and its vasoregulatory effects occur through both endothelium-dependent and endothelium-independent mechanisms. TNF-α promotes vasodilation by increasing the production of nitric oxide, hydrogen peroxide, and prostaglandins. Conversely, TNF-α induces vasoconstriction by elevating the levels of endothelin-1 and angiotensinogen. Additionally, excess TNF-α could impair endothelium-dependent vasodilation in various vascular beds by enhancing reactive oxygen species (ROS) production and subsequently increasing nitric oxide scavenging [255]. TNF-α can also induce the production of chemerin from adipocytes [257]. Chemerin is an endogenous substance that causes vasoconstriction by promoting eNOS uncoupling, which reduces nitric oxide production and shifts toward generating ROS [258]. Another example is omentin-1, which has been linked to vasodilation [259] by promoting nitric oxide production in endothelial cells, maintaining appropriate blood flow, and lowering cardiovascular risk [260]. Other examples of adipokines that exhibit vasoactive properties by promoting the release of ROS are IL-6, resistin, visfatin, apelin, and adipose-derived relaxing factor (ADRF) [255].

The vascular-adipose relationship is bidirectional. For example, obesity and metabolic diseases are accompanied by vascular endothelial dysfunction, which leads to hypoxia and a reduced blood supply to adipose tissues. In our recent research, we have identified hypoxic adipocytes and induced hypoxia-inducible factor 1 alpha (HIF1α) as the driving factor for various epigenetic perturbations that alter the secretory role of adipose tissue. Primarily, we determined the role of hypoxia in promoting the activity of the DNA hydroxymethylase, TET1, which results in global and gene-specific DNA hypomethylation. Downstream targets for this mechanism include inflammatory adipokines such as resistin, leptin, visfatin, and IL-6. This increased release of adipokines had a detrimental effect on vascular function within the adipose tissue and at a distance in the major arteries [215,261,262].

Hypoxia within the WAT was also found to reduce adiponectin secretion, contributing to reduced EV secretion from endothelial cells. This cascade of events can expedite the progression of endothelial dysfunction and exacerbate the decline in adipocyte function [263]. Additionally, the absence of adiponectin results in mitochondrial impairment, endothelial activation, and disrupted pulmonary vasculature. This mechanism engages PGC-1α and its downstream effectors, such as nuclear respiratory factor 1, transcription factor A, mitochondrial, Sirtuin (Sirt)3, and Sirt1 expression [264].

### 6.3. Adipose Tissue–Metabolic Axis

The adipoinsular axis is a term that refers to the interaction between pancreatic beta cells and adipokines, which is essential for the preservation of metabolic equilibrium. Adipokines impact the activity and viability of pancreatic beta cells, whereas the insulin secreted by these cells affects the metabolism of adipose tissue, hence impacting the storage and breakdown of lipids. Prolonged insulin resistance and obesity can trigger the release of excessive amounts of fatty acids and inflammatory compounds by adipose tissue, thereby contributing to the malfunction and apoptosis of beta cells. These factors eventually result in the development of T2DM [265].

Glucose-stimulated insulin secretion by beta cells is impacted by adipokines, leading to various effects on insulin secretion. Leptin, for instance, inhibits insulin secretion under normal physiological conditions through intricate mechanisms that involve the modulation of potassium channels, intracellular signaling pathways, and apoptosis regulation. On the other hand, adiponectin enhances insulin sensitivity, safeguards beta cells, and activates glucose-stimulated insulin secretion. Additional adipokines such as apelin, resistin, visfatin, and others influence beta cell function and insulin secretion. Apelin, for instance, plays a role in maintaining beta cell homeostasis, and its deficiency can lead to impaired glucose metabolism. Resistin appears to impact beta cell apoptosis and the expression of insulin receptors. Visfatin exhibits insulin-mimetic properties and was found to be associated with insulin secretion, beta-cell proliferation, and cell viability. Other adipokines like adipsin, lipocalin-2, chemerin, FGF21, GDF15, and TNF-α contribute to the intricate network of interactions between adipokines and beta cells. These adipokines have been observed to affect beta cell mass, insulin secretion, and overall metabolic regulation [265].

Vital metabolic organs like the liver and skeletal muscles interact with adipose tissues to maintain the body’s homeostasis. Adipokines, myokines, and hepatokines work in synergy within the body, creating a complex series of actions across various tissues. Any disturbances in this network will negatively impact different physiological functions, predisposing to several metabolic and CVDs [266]. Myostatin is a myokine/adipokine that has pro-inflammatory properties. Its levels tend to rise in conditions characterized by physical inactivity and increased fat mass [267]. Myostatin decreases insulin receptor phosphorylation, contributing to the development of hyperglycemia. It also promotes increased glucose uptake by the liver and leads to the accumulation of fat in the liver, a condition known as hepatic steatosis [268,269]. Myostatin inhibits the release of myokines and other organokines that possess anti-inflammatory and antioxidant properties while simultaneously promoting adiposity and the release of inflammatory adipokines and hepatokines. This imbalance in the secretion of various signaling molecules further contributes to the chronic inflammatory state associated with obesity and other cardiometabolic diseases [270].

Consequently, leptin, TNF-α, myostatin, chemerin, resistin, fetuin-A, and other inflammatory adipokines collectively promote oxidative stress and inflammation characterized by an increase in M1 macrophages within adipose tissue, heightened production of ROS, and impaired insulin action [268]. This pro-inflammatory secretory pattern also influences the hemodynamic patterns and vascular endothelial function, further increasing cardiovascular risk. When combined with factors like visceral obesity, dyslipidemia, and hyperglycemia, this pattern creates a scenario characteristic of metabolic syndrome [216,271]. Hence, myostatin is recognized as a potential therapeutic focus for addressing and controlling these metabolic disorders [269]. Similarly, persistent elevation of IL-6 and TNF-α, released by adipocytes and muscle cells, can lead to the progression of insulin resistance and subsequently contribute to the onset of metabolic diseases [267]. In obese and diabetic individuals, macrophages in visceral adipose tissue produce TNF-α and other pro-inflammatory cytokines that impair insulin action in skeletal muscles and initiate signaling pathways that impair muscle strength and induce muscle atrophy. In addition, obesity-associated hyperliptinemia and leptin resistance hamper muscle fatty acid oxidation and decrease lipolysis [267,268].

FGF21 is a prototype of an adipokine that plays a vital role in regulating lipid and glucose metabolism. It exerts its effects by controlling insulin sensitivity through various mechanisms. Besides stimulating thermogenesis and promoting the generation of heat and energy expenditure in situ in the adipose tissue, FGF21 enhances fat oxidation and promotes the use of fatty acids as an energy source in skeletal muscles [272,273]. FGF21 functions as a regulator of beta-oxidation, the mechanism by which fatty acids are broken down and used for energy production. In the liver, FGF21 inhibits de novo lipogenesis, thereby reducing the production of lipids. These effects are particularly prominent when FGF21 levels are increased during the post-exercise period [268]. Clinical studies on patients with T2DM have shown that FGF21 analogs reduce LDL while increasing HDL levels. Additionally, FGF21 analogs improve insulin action, leading to better glycemic control and reduced cardiovascular risks. These analogs have also demonstrated positive effects on body weight regulation and have been associated with increased adiponectin and its insulin-sensitizing properties [274,275].

By disrupting the balance of circulating free fatty acids and inflammatory cytokines, dysfunctional adipose tissues in the context of obesity and metabolic disorders contribute to liver triglyceride (TG) accumulation. For instance, the augmented production of resistin has been associated with inflammatory cell infiltration in the liver and an increased risk of non-alcoholic steatohepatitis (NASH) [276]. The protective adipokine adiponectin, on the other hand, binds to its liver receptors, AdipoR1 and AdipoR2, suppressing critical gluconeogenic regulators such as phosphoenolpyruvate carboxykinase and glucose-6-phosphatase. By blocking these enzymes, adiponectin decreases glucose synthesis in the liver, lowering blood glucose levels [277,278].

The adipokines and hepatokines of adipose tissue and the liver facilitate crosstalk between these two organs. For example, follistatin, a hepatokine released in higher amounts during exercise, neutralizes myostatin and counteracts its pro-inflammatory effects, aiding in the breakdown of stored fats in WAT. Additionally, research has shown that follistatin, in conjunction with irisin (a myokine produced during exercise), protects the function of pancreatic beta cells [268]. However, in diabetic patients, the circulating follistatin levels were increased, exhibiting correlations with markers of insulin resistance, including fasting glucose, glucose tolerance, and glycosylated hemoglobin (HbA1c) [279]. Another example is adropin, a hepatokine that enhances glucose uptake by metabolic tissues via activating glucose transporters. It also enhances glucose utilization by sensitizing metabolic tissues to insulin through the induction of insulin signaling pathways, including Akt phosphorylation. Adropin inhibits inflammation and improves lipid profiles by reducing LDL and total cholesterol while elevating HDL levels. It also improves cardiac function and blood flow by enhancing nitric oxide production. Furthermore, adropin stimulates the expression of fibronectin and elastin in VSMCs by modulating the PI3K-Akt pathway [280]. Other hepatokines such as fetuin-A, hepassocin, LECT2 (leukocyte cell-derived chemotaxin-2), and selenoprotein are commonly increased in obesity and result in the systemic inflammatory state [281]. Fetuin-A and -B have been associated with adverse effects on metabolism and insulin sensitivity. They also impact β-cell function and increase lipotoxicity by activating the TLR4-JNK-NF-κB pathway [282].

The examples above highlight the role of soluble mediators and adipokines in mediating the interplay between adipose tissue and other metabolic organs. Apart from these soluble mediators, those carried inside EVs play a crucial role in mediating this crosstalk. Previous studies have shown that the hypoxic environment in adipose tissues alters the EV protein cargo. Proteomic analysis of EVs produced by adipocytes cultured under hypoxic conditions revealed 75 upregulated proteins and 67 downregulated proteins compared to those cultured under normoxia. Furthermore, the EVs from hypoxic adipocytes were enriched with de novo lipogenesis proteins, significantly promoting lipid accumulation in recipient cells [253].

On the other hand, EVs derived from liver cells were found to alter lipid metabolism in adipose tissues. In mice fed a high-fat diet (HFD), there is a rapid onset of lipid accumulation in the liver, occurring within hours. In response to this lipid overload, the liver increases its secretion of EVs, specifically targeting adipocytes [283]. Liver-derived EVs enhanced lipid deposition in adipocytes by promoting lipogenesis and inhibiting lipid oxidation, likely through the modulation of PGC1α (a transcriptional coactivator involved in mitochondrial biogenesis and energy metabolism). Inhibiting liver generation of EVs, through knockdown of Geranylgeranyl diphosphate synthase (*Ggpps*), improved glucose tolerance and lipid composition in the adipose tissues of HFD-fed mice. These findings suggest that liver cells may act as early metabolic sensors of lipid overload and respond by increasing EV signaling to adipocytes [283].

### 6.4. Adipose Tissue–GIT Axis

Over the past several years, there has been growing recognition of the role of intestinal microorganisms in regulating various metabolic functions, including the secretion of adipokines. Evidence suggests that changes in the composition and function of the gut microbiota, known as dysbiosis, can contribute to the development of metabolic conditions such as obesity, T2DM, liver disease, cancer, and even neurological disorders. Changes in the gut microbiota composition have been associated with a decrease in the thickness of the protective mucus layer, disruption of tight junction proteins, and decreased release of antimicrobial peptides. These changes can lead to the translocation of pathogen-associated molecular patterns (PAMPs), which can induce abnormal immune responses and chronic low-grade inflammation in the host [284]. Dysbiosis, characterized by an imbalance in the gut microbiota, has been associated with various metabolic changes. One such change is modifications in bile acid profiles, where the composition of bile acids is altered.

Additionally, dysbiosis is associated with decreased secretion of gut peptides, which play essential roles in regulating appetite and satiety. There is also a decrease in short-chain fatty acids (SCFAs), which are helpful metabolites produced by gut bacteria through the fermentation of dietary fibers. Conversely, dysbiosis is often accompanied by higher levels of branched-chain amino acids (BCAAs) [285]. Indeed, SCFAs can bind to specific G protein-coupled receptors (GPCRs) located on various cells within the gastrointestinal tract. This binding leads to the activation of these receptors, which subsequently triggers the release of several essential gut peptides, including GLP-1, glucagon-like peptide 2 (GLP-2), and peptide YY (PYY) [286].

The hormones PYY, pancreatic polypeptide (PP), oxyntomodulin (OXM), gastric inhibitory polypeptide (GIP), cholecystokinin (CCK), and GLP-1 derived from the gastrointestinal tract (GIT) function as factors that reduce food intake and boost energy expenditure. GIT hormones are typically released after meals, and their secretion is inhibited during fasting or prior to meals, except for ghrelin, which stimulates appetite and food intake [287]. Gut hormones can influence the secretion of adipokines, enabling them to detect nutrient availability and regulate peripheral metabolism and metabolic status. Administration of amylin, GLP-1, OXM (oxyntomodulin), and CCK (cholecystokinin) has been demonstrated to stimulate the secretion of adiponectin [288]. Ex vivo studies that used human adipose tissue explants showed a positive effect of amylin treatment on leptin signaling mediated by activating a G-protein-coupled receptor (GPCR) [289]. In animal models, the administration of CCK and OXM has been shown to decrease leptin levels [290,291]. The administration of pancreatic polypeptide (PP) to food-deprived mice has also been shown to reduce leptin expression in WAT; this effect was thought to involve, at least partially, the sympathetic innervation of adipose tissue [292]. Sitticharoon et al. [217] have shown that visfatin expression in visceral adipose tissue is inversely correlated with serum levels of PYY in human subjects, suggesting a potential protective effect and a possible regulatory role of PYY in adipose tissue metabolism.

Furthermore, the administration of either GIP or a long-lasting analog has been shown to decrease the inflammatory profile of the visceral adipose tissue secretome while elevating adiponectin secretion [293,294]. Additionally, GLP-1 has been found to reduce the production of nuclear factor kappa B (NF-κB)-mediated pro-inflammatory adipokines such as interleukin-6 (IL-6), TNF-α, and monocyte chemoattractant protein-1 (MCP-1) in visceral adipose tissue [295]. Similarly, acylated and de-acylated ghrelin have been suggested to exhibit an anti-inflammatory role by reducing TNF-α-induced apoptosis in human adipocytes. This effect is achieved by inhibiting caspase-8 and caspase-3, key enzymes involved in the apoptotic pathway [296].

In addition to modifying adipokine production, GIT hormones regulate several metabolic functions in the adipose tissues. For instance, ghrelin induces the activation of SREBP1c, leading to a rise in the expression of lipoprotein lipase (LPL) and lipogenic enzymes. Additionally, ghrelin decreases the outflow of glycerol [297]. These effects preserve energy stores in WAT during periods of starvation. GIP, Amylin, and CCK may exert similar effects to ghrelin. Amylin, for instance, enhances the insulin effect in adipocytes, resulting in decreased glycerol release and increased incorporation of fatty acids [298]. In the presence of insulin, GIP enhances insulin action by promoting fatty acid uptake [299]. Moreover, during physiological postprandial conditions, GIP increases hydrolysis of circulating triglycerides (TAGs) and adipocyte re-esterification, reducing the outflow of fatty acids. Furthermore, GIP stimulates the expression of the *LPL* gene through multiple pathways that involve the activation of Akt, 5′ AMP-activated protein kinase (AMPK), and cAMP response element binding (CREB)-dependent transcription [300]. Similar to GIP, CCK-8 (cholecystokinin-8) enhances the activity of LPL in adipose tissue through a mechanism that relies on the CCK-2 receptor [290]. GLP-1 acts as a factor that promotes lipolysis and inhibits lipogenesis in human adipose tissue (AT) and cultured adipocytes. When the GLP-1 receptor is activated through a cAMP/protein kinase A (PKA) pathway, perilipin undergoes hyperphosphorylation, leading to the mobilization of fat and increased exposure of adipose tissue lipases (ATGL), which further contributes to the process of lipolysis [301].

Recent research indicates that the gut microbiome can influence the host’s metabolism through cross-talk with adipose tissue. It can modulate adipogenesis and energy expenditure and help reduce obesity by promoting the conversion of WAT to BAT and increasing BAT activity. Moreover, the gut microbiome is an important environmental factor that regulates the profile of adipokines. These adipokines can, in turn, interfere with the functional changes in adipose tissue caused by the microbiome. On the other hand, adipokines like leptin, adiponectin, FGF21, and apelin can also act on the gut microbiome to alter its composition [302]. Previous studies have identified a population of perivascular cells within the intestinal submucosa expressing the leptin receptor (LepRb). Signaling through these LepRb receptors has been shown to regulate the host’s gut microbiome composition [303]. It has also been shown that daily supplementation of leptin causes a more significant proportion of *Clostridium* genus and *Sutterella* and enhances the expressions of TNF-α and mucin (MUC-2 and MUC-3) during the suckling period of rats, indicating a modulator role of leptin on the intestinal activation [304].

Similarly, adiponectin has been demonstrated to impact the diversity of microbial species in the gut microbiome [304]. Adiponectin deficiency has been linked to changes in the gut microbiome, particularly the bacterial genera *Prevotella*, *Bacteroides*, and *Helicobacter* implicated in energy homeostasis and inflammatory bowel diseases [305]. Daily supplementation with adiponectin in animal studies caused a lower relative population of bacteria in the *Proteobacteria* phylum, the Blautia and Roseburia genera, and more *Enterococcus* genus bacteria. In addition, providing supplemental adiponectin to rats during the suckling stage was observed to modify the composition of the gut microbial community and enhance immune system functions [304]. On the other hand, certain microbiome species exert their function through adiponectin signaling. For instance, supplementation with the flavonoid compound from *Smilax china* L. (SCF) was found to alleviate weight gain, fat accumulation, abnormal serum lipid levels, hepatic steatosis, and improved glucose regulation. These metabolic effects of SCF were suggested to be mediated by upregulation of the adiponectin receptor/AMPK signaling pathway, which in turn enhances the gut microbiome composition [306].

Genetically obese and type 2 diabetic mice exhibit substantial alterations in their gut microbial makeup compared to lean controls, which is believed to be partially attributed to modifications in the apelinergic system in the adipose tissues. In type 2 diabetic mice, modified gut microbiome composition, characterized by increased abundance of the *Firmicutes*, *Proteobacteria*, and *Fibrobacteres* phyla, modulates the expression of apelin and its receptor APJ in adipose tissue via low-grade inflammatory state and the endocannabinoid system [307]. Supplementation with prebiotics or probiotics is widely regarded as a practical and effective approach to promote energy homeostasis [285]. Studies have shown that prebiotics can restore leptin sensitivity in rodents with HFD-induced obesity and diabetes, suggesting that targeting the gut microbiota can potentially restore adipokine homeostasis [286,308].

### 6.5. Adipose Tissue–Immune Cells

Adipocytes play a role in the adaptive immune system by recruiting and activating immune cells via adipokines such as leptin, adiponectin, and resistin [309]. These adipocytes act as antigen-presenting cells (APC), expressing MHC class I and II molecules and costimulatory molecules CD80/CD86 [310]. Through this role, adipocytes provide the three signals required for T-cell activation and differentiation: antigen presentation, costimulation, and cytokine stimulation. Additionally, adipocytes regulate the differentiation and function of T-cells and B-cells through the secretion of cytokines, such as leptin, resistin, TNF-α, IL-2, IL-4, IL-6, and interferon-gamma (IFNγ) [311,312].

This immune function of adipocytes is dysregulated in metabolic diseases, contributing to the associated inflammation. For example, in obese HFD-fed mice, adipocyte expression of MHC II molecules had increased within two weeks. Also, the T-Box Transcription Factor 21 (*Tbx21*) and *IFNγ* genes, markers for the pro-inflammatory Th1 (*T helper 1 cells*) cells within adipose tissue, had increased two to three folds [313]. Furthermore, in response to HFD, mice deficient in adipocyte MHC II molecules have shown an increase in regulatory T-cells (Treg) in visceral adipose tissue (VAT), lower levels of inflammation, and higher insulin resistance compared to wild-type mice [314].

An example of the adipokines that possess pro-inflammatory functions is leptin, which promotes the creation of naïve and memory T-cells, increasing the production of Th1 cytokines while suppressing the production of Th2 cytokines and preventing Treg activation [315]. The significance of leptin in the production of B-cells was demonstrated in leptin-deficient rodents, which exhibited a low number of B-cells restored by leptin injection [316]. IL-6 is another example of pro-inflammatory adipokines that promote T-cell differentiation, increasing the body’s immune response and inflammation. Furthermore, IL-6 has also been shown to promote inflammation by preventing TGF-β from promoting Treg cell differentiation, interfering with the creation of anti-inflammatory cells [317,318].

Additionally, other adipokines produced by adipocytes, such as resistin, visfatin, and TNF-α, also regulate T-cell function. Resistin has been shown to suppress the interferon regulatory factor-1, regulate dendritic cells, and enable the production of Treg cells, inhibiting T-cell production and preventing an increase in inflammation [319]. Visfatin, on the other hand, is an adipokine that increases the activation of T-cells through the increased production of pro-inflammatory cytokines such as IL-1β, IL-6, and IL-10 and the expression of costimulatory molecules such as CD80 and CD40, resulting in an augmented immune response and inflammation [320]. Moreover, studies have shown that TNF-α is secreted by activated adipocytes alongside other immune cells and is vital to regulating T-cells, memory T-cells, and the body’s immune response sensitivity [321,322].

Similar to leptin, adiponectin also has receptors on the surface of B-cells [323] and has been shown to prevent inflammation by encouraging B-cells to produce the PEPITEM peptide. This peptide suppresses helper and memory T-cell migration [324], thereby preventing the T-cells from traveling throughout the body and triggering an immune response and inflammation. On the other hand, the impact of adiponectin on T-cells is controversial. Adiponectin can either promote or suppress T-cell differentiation and inflammation. Studies have shown that adiponectin promotes apoptosis of T-cells [325], preventing the secretion of pro-inflammatory cytokines and inhibiting an increase in inflammation. However, other studies have also demonstrated that adiponectin increases T-cell activation, which in turn increases inflammation by amplification of the Th17 and T follicular helper (Tfh) cell response in collagen-induced arthritis [326] and by increasing the expression of B-cell lymphoma-extra large (Bcl-xL) and B-cell lymphoma 2 (Bcl-2), anti-apoptotic proteins, in IBD patients [327], enhancing T-cell production and inflammation.

Visceral adipose tissue (VAT) contains a substantial population of immune cells. Most of these cells exhibit an anti-inflammatory or T-helper 2 (Th2) nature. One vital subset of immune cells responsible for maintaining adipose tissue’s immune balance is adipose tissue macrophages (ATMs). In lean conditions, ATMs assume an M2 phenotype, which involves the synthesis of anti-inflammatory substances like IL-10 and IL-1 receptor alpha (ILRα) [328]. M2 ATMs rely on Th2 cytokines produced by various sources, including immune cells, adipose tissue stroma, and adipocytes, for continued activation. This active maintenance is crucial for sustaining the alternatively activated state of M2 ATMs [329].

Adipocytes play a central role as immune cell subsets within VAT, exerting control over the activity of local immune cells. Furthermore, specialized immune cell subsets contribute to maintaining adipose tissue homeostasis. Among these, CD4^+^ T-cells represent the predominant T-cell subset within VAT, with a significant proportion of these cells being regulatory T-cells (Tregs) expressing the transcription factor FoxP3. This distinctive composition of CD4^+^ T-cells sets VAT apart from other tissues [330]. Studies have demonstrated the essential role of IL-10, produced by Tregs, in preventing inflammation within VAT [330]. The survival of Tregs within VAT relies explicitly on the cytokine IL-33 produced by the stromal cells of adipose tissue [330]. Obesity-associated reductions in IL-33 expression in VAT impair the function of Tregs and dysregulate the immune response, leading to an increased susceptibility to developing T2DM [330].

Another crucial mechanism for maintaining adipose tissue homeostasis is the IL-33/ILC2/eosinophil axis. Apart from supporting the survival of regulatory T-cells, IL-33 derived from adipose tissue stroma is essential for maintaining type 2 innate lymphocytes (ILC2s). IL-33 ensures the proper functioning and survival of ILC2s, which are key regulators of immune responses within adipose tissue. This axis is vital in balancing immune cell populations and sustaining the immune environment necessary for adipose tissue homeostasis [330]. ILC2s are the primary source of IL-5 within adipose tissue, which is crucial for the maintenance and survival of eosinophils [330]. In response to certain signals, eosinophils produce IL-4 and, to a lesser extent, IL-13 within adipose tissue, essential for driving the alternative activation of adipose tissue macrophages (ATMs). IL-4 and IL-13 act as key regulators that promote the shift of ATMs from a pro-inflammatory M1 phenotype to an anti-inflammatory M2 phenotype. This alternative activation is associated with tissue remodeling, inflammation resolution, and maintaining adipose tissue homeostasis [330]. The disruption or removal of any component within the IL-33/ILC2/Eosinophil axis leads to heightened adipose tissue inflammation and facilitates the development of insulin resistance in response to diet-induced obesity (DIO) [330]. Therefore, the interactions between adipocytes and immune cells play a crucial role in orchestrating immune responses and maintaining a balanced immune environment within adipose tissue under healthy body weight conditions, helping to regulate metabolic processes, control inflammation, and preserve tissue functionality.

Obesity is characterized by circulating chronic inflammatory molecules that significantly activate the immune system, particularly within adipose tissue [330]. As obesity progresses, a substantial number of pro-inflammatory ATMs are recruited into VAT, causing a shift in the ATM phenotype from the anti-inflammatory M2 phenotype to the pro-inflammatory M1 phenotype [330]. Leptin, a hormone predominantly secreted by adipose tissue, is believed to be a significant factor contributing to the accumulation of immune cells in obese adipose tissue, as leptin acts as a potent stimulator, promoting the proliferation and activation of immune cells. Therefore, increased leptin levels observed in obesity can influence the immune cell population within adipose tissue, potentially leading to an enhanced immune response and inflammation [330]. Initially, immune cells infiltrating adipose tissue in response to HFD do not display an overt pro-inflammatory phenotype. Instead, immune cell-derived cytokines released in response to adipocyte stress play a crucial role in macrophage activation and the subsequent development of inflammation in obese VAT. These cytokines contribute to the induction of macrophages and the initiation of an inflammatory response within the VAT in the context of obesity [330]. When adipocytes undergo hypertrophy, they are subjected to various stressors, including microhypoxia, endoplasmic reticulum stress, and extracellular matrix confinement [330]. Under the influence of the aforementioned stressors, adipocytes upregulate stress ligands on their surface. Among these molecules, a specific ligand interacts with the NK cell, activating receptor NKp46. In obese VAT, the engagement of NKp46 with its ligand leads to the activation of NK cells, triggering their proliferation, representing an immune response to the stressed adipocytes in obesity [330].

A notable characteristic of NK cells in obese VAT is their ability to produce IFNγ, which facilitates the stimulation of macrophages and promotes their pro-inflammatory phenotype within VAT, contributing to the inflammatory environment observed in obese adipose tissue [330]. In addition to NK cells, CD8^+^ T-cells have been suggested as a potential source of factors that support the activation and accumulation of ATMs in obese VAT. The population of CD8^+^ T-cells increases in response to HFD, contributing to the immune cell composition seen in the VAT of obese individuals. These CD8^+^ T-cells may release factors that create a viable environment for the activation and maintenance of ATMs, further exacerbating inflammation within the adipose tissue [330]. Invariant-chain NKT (iNKT) cells are an additional subset of T-cells that regulate immune cell function under normal physiological conditions. Within adipose tissue, iNKT cells operate as the primary source of IL-13, a cytokine that strongly contributes to maintaining an alternatively activated macrophage (AAM) phenotype in ATMs [330]. However, in obesity, iNKT cells produce excess IFNγ and induce an increase in insulin resistance. As the inflammatory process progresses, regulatory cells (Tregs), essential for maintaining immune homeostasis and limiting excessive inflammation, are lost. This reduction in Tregs occurs alongside an increased abundance of pro-inflammatory Th1 cells and NKT cells, further contributing to the chronic inflammation and metabolic dysregulation associated with obesity [330].

Adiponectin has been found to suppress the production of TNF-α and the expression of TNF-α mRNA induced by LPS stimulation [331], inhibiting adipose tissue inflammation. Additionally, adiponectin has been demonstrated to increase the transcript levels of M2 phenotype markers, such as IL-10, suggesting that it facilitates the polarization of macrophages toward an anti-inflammatory M2 phenotype [332]. In support of these assumptions, previous studies on mature human macrophages have demonstrated that adiponectin treatment inhibits their phagocytic activity.

Adipolin, alternatively called C1q/TNF-related protein 12, is an adipokine enhancing insulin sensitivity [332]. Deficiency of adipolin leads to enhanced inflammatory responses, increased vascular cell proliferation, and reduced levels of TGF-β 1 protein in injured arteries. Cultured macrophages treated with adipolin protein result in decreased expression of inflammatory mediators, such as TNF-α, interleukin (IL)-6, and monocyte chemotactic protein (MCP)-1 stimulated by LPS. Adipolin also reduces the proliferation of VSMCs stimulated by platelet-derived growth factor (PDGF)-BB through a TGF-βRII/Smad2-dependent pathway. Treatment with adipolin has been shown to protect against pathological vascular remodeling by attenuating macrophage inflammatory responses and VSMC proliferation [332].

### 6.6. Adipose Tissue–Bone Axis

Adipose tissue secretes a range of adipokines, such as adiponectin, leptin, interleukin-6 (IL-6), tumor necrosis factor-α (TNF-α), resistin, and visfatin, which play critical roles in the intricate control of bone functions. On the other hand, bone has been recognized as an endocrine organ capable of influencing body weight, energy utilization, and glucose balance [333]. Leptin exerts a dual influence on bone health. One beneficial aspect involves its ability to promote stromal cell transformation into osteoblasts, boost their proliferation, and impede the formation of osteoclasts. Accordingly, lacking the leptin gene leads to diminished bone mineral density (BMD) and bone volume [334]. Despite evidence supporting the positive correlation between leptin and BMD, adverse effects have also been documented [335]. This negative impact is believed to occur through the central nervous system, where leptin can reduce serotonin production in hypothalamic neurons, subsequently hindering bone formation. Mice lacking leptin or its receptors demonstrated femur bone mass reductions and bone marrow fat increases [334].

Similar to leptin, adiponectin boosts the proliferation of osteoblasts, elevating alkaline phosphatase activity and facilitating the production of type I collagen and osteocalcin, all of which serve as indicators of osteoblast differentiation and maturity. Adiponectin-induced osteogenesis in mesenchymal stem cells operates through adipoR1 phosphorylation of P38 MAPK, which amplifies cyclooxygenase-2 (COX-2) and bone morphogenic protein 2 (BMP2) expression. IL-6 and TNF-α, on the other hand, prompt osteoclast formation and bone resorption via several mechanisms that trigger an increase in receptor activator of nuclear factor kappa B ligand (RANKL), a factor that propels osteoclastic bone resorption [334]. Apelin is another adipokine that aids in regulating bone metabolism. Apelin-13 potentially safeguards bone health by influencing inflammatory pathways and programmed cell death in bone marrow stromal cells, processes crucial for bone strength and osteoblast formation, and preventing their breakdown [336]. Conversely, resistin’s impact on bone remains contentious. While it promotes osteoblast proliferation, it also supports osteoclast proliferation and the secretion of inflammatory cytokines [334].

Chemerin, produced by the WAT, is implicated in bone health. The absence of chemerin correlates with heightened gene expression associated with osteoblast formation and the suppression of genes related to osteoclast development pathways [337]. In a knockout mouse model where chemerin protein or its receptor (chemokine-like receptor 1; CMKLR1) is absent, enhancements in bone fortification mechanisms were observed. These enhancements involved the increased expression of osteoblast marker genes and bone mineralization when exposed to stimuli that promote osteoblast formation. Chemerin/CMKLR1 has also been found to modify the expression of specific transcription factors linked to osteoclast development, thereby restricting the osteoblast-promoting Wnt signaling pathway [338].

Other adipokines implicated in bone health include lipocalin-2, Metrnl, omentin-1, and visfatin. For lipocalin-1, excessive protein production and heightened gene expression prompt the production of RANKL and IL-6. Consequently, this stimulation leads to osteoclast formation and inhibits the maturation of osteoblasts [339]. METrnl was shown to enhance osteoblast function, which is likely mediated by its effect on macrophages [340]. Similarly, omentin-1 preserves optimal bone density, mitigates inflammation, and prevents bone loss. These effects are likely achieved through the suppression of pro-inflammatory agents. Omentin-1 can hinder the inflammatory reactions triggered by activated macrophages and diminish their anti-osteoblastic and pro-osteoclastic functions [341]. Finally, visfatin was engaged in inflammation and bone breakdown, notably affecting glucose uptake and collagen synthesis in osteoblasts [342].

The compounds secreted from bones, called osteokines, also influence the functions and mass of fat tissue. Osteocalcin (OCN), primarily released by osteoblasts, diminishes starvation, malnutrition, and anorexia nervosa. OCN is traditionally regarded as a biological indicator of bone formation, and its deficiency in mice correlated with heightened body fat and insulin resistance. OCN demonstrates the capacity to enhance glucose tolerance in living organisms by promoting insulin expression and β-cell growth, along with inducing adiponectin expression and activating genes responsible for energy expenditure in adipocytes [333]. In WAT, OCN enhances insulin sensitivity by reducing inflammation, boosting insulin signaling, and elevating the expression of glucose transporter type 4 (GLUT4) [343]. Osteopontin (OPN) is another osteokine produced by osteoclasts, adipocytes, and macrophages associated with T2DM and obesity. Additionally, OPN is proposed to have a crucial function in connecting obesity with the onset of insulin resistance by encouraging inflammation and the buildup of macrophages in adipose tissue [344].

Osteoprotegerin (OPG), along with RANKL and its receptor RANK, regulates bone breakdown. Remarkably, OPG and RANKL are present in various regions of rat adipose tissue, and their expression rises during the differentiation of adipocytes [345]. Elevated levels of OPG in mice fed a high-fat diet result in high blood sugar levels, an upsurge in inflammatory cytokines, and the accumulation of macrophages in adipose tissue [346]. OPG undergoes a significant increase during obesity progression, and its expression is triggered by high blood sugar and increased insulin levels in adipocytes within visceral fat tissue at the early stages of obesity development, leading to adipocyte enlargement and internal lipid accumulation. Cellular OPG can bind to RAR, releasing PPARɤ/RXR and activating the transcription of adipogenesis genes. The elevated levels of OPG play a crucial role in adipocyte maturation and lipid retention. OPG deletion, specifically in adipose tissue, delays obesity onset, whereas OPG supplementation accelerates obesity advancement and insulin resistance [347].

### 6.7. Adipose Tissue–Cardiac Axis

An essential factor in preserving cardiometabolic health is the interaction between cardiokines and adipose tissue. Bioactive chemicals generated by the heart, known as cardiokines, influence systemic metabolism. This reciprocal interaction between the heart and adipose tissue affects numerous physiological functions, including cardiovascular health, insulin sensitivity, inflammation, and energy balance.

Numerous studies have shown that adipokines can have either beneficial or harmful impacts on cardiac functions. Alterations in the levels of adipokines like adiponectin, lipocalin-2, omentin-1, vaspin, and leptin play a role in the development of heart failure [348]. Leptin mitigates myocardial reperfusion-induced damage and helps the heart to compensate for myocardial cell elongation and eccentric dilation. However, excess leptin in obese individuals enhances atherosclerosis by stimulating oxidative stress and cholesterol absorption [349]. Furthermore, increased heart rate and blood pressure caused by leptin might lead to an increased burden on the heart muscle, promoting long-term cardiac hypertrophy by stimulating the sympathetic nervous system. Moreover, leptin might impact cardiomyocyte enlargement through diverse pathways, pro- and anti-hypertrophic [350].

Adiponectin is another adipokine known for its cardioprotective effects. Sufficient adiponectin levels have been linked to maintaining proper cardiomyocyte functionality and safeguarding against cardiac enlargement, oxidative stress, and inflammation [351]. Reduced adiponectin levels are linked to conditions such as left ventricular hypertrophy, whereas adequate levels decrease the likelihood of ischemic events in men [352]. Similarly, apelin-13 has been shown to positively impact cardiac function in rats with heart failure by ameliorating cardiac dysfunction, alleviating compromised hemodynamics, and decreasing fibrosis and oxidative stress [353].

The adipokine resistin, which is released by visceral fat, plays a role in insulin resistance and chronic inflammation, therefore exacerbating cardiovascular health. Elevated levels of resistin facilitate the development of atherosclerosis and hinder the activity of cardiokines such as B-type natriuretic peptide (BNP), exacerbating metabolic and cardiovascular disorders [354]. Circulating chemerin has also been associated with metabolic syndrome, coronary artery disease, and inflammation. Serum chemerin is a novel and valuable indicator for predicting significant adverse cardiac events in individuals with congestive heart failure (CHF). The detection of circulating chemerin can improve the early recognition of CHF patients at risk [355]. Interleukin-6 (IL-6), produced by adipose tissue, has a dual function in the functioning of cardiomyocytes. It protects the cardiac tissues during the acute phase response by stimulating immune cell responses. However, prolonged activation of IL6 leads to persistent inflammation and fibrosis, resulting in impaired myocardial contractility and ventricular dysfunction in human subjects [356].

On the other hand, cardiokines contribute to the critical communication network between the heart and adipose tissue, impacting cardiovascular and metabolic health. Key cardiokines include BNP, ANP (atrial natriuretic peptide), FGF21, adrenomedullin (ADM), angiotensin II (Ang II), atrial natriuretic factor (ANF), tumor necrosis factor-alpha (TNF-α), and follistatin-like (FSTL) 1. These cardiokines regulate several functions, such as cellular growth, remodeling, apoptosis, mitochondrial function, and several aspects of metabolism [357]. BNP and ANP bind to natriuretic peptide receptors (NPR-A) in adipose tissue, which leads to the activation of lipolysis [358]. These cardiokines also stimulate the browning of WAT by increasing mitochondrial biogenesis and promoting thermogenesis, similar to BAT [359]. Through these actions, BNP and ANP contribute to weight loss, improved insulin sensitivity, increased energy expenditure, and lowered risk of obesity and metabolic syndrome [360]. Adipose tissue, in turn, regulates BNP and ANP through adipokines. For instance, adiponectin enhances the sensitivity of cardiomyocytes to natriuretic peptides, facilitating cardioprotection [361].

FGF21 is another cardiokine that enhances the browning of WAT and increases fatty acid oxidation, improving overall energy expenditure [362]. It also increases insulin sensitivity and helps regulate glucose homeostasis. FGF21 exerts anti-obesity, anti-diabetic, and anti-inflammatory effects by enhancing lipid and glucose metabolism. Its role in reducing triglycerides and enhancing glucose uptake helps mitigate the risk of CVD and metabolic disorders [134]. In turn, adipose tissue produces adipokines like adiponectin, which can stimulate cardiac FGF21 production and improve cardiac metabolism [363].

The cardiokine adrenomedullin (ADM) regulates lipolysis and enhances insulin sensitivity in adipose tissue, contributing to overall metabolic homeostasis [364]. It also has anti-inflammatory effects on adipose tissue, reducing the secretion of pro-inflammatory adipokines such as TNF-α and IL-6 [365,366]. ADM helps improve vascular function, reduce hypertension, and promote insulin sensitivity—all factors that reduce the risk of metabolic syndrome and cardiovascular disease [367]. By improving insulin sensitivity and reducing inflammation, ADM protects the heart from metabolic stress and hypertrophy, enhancing cardiovascular function [368]. In conclusion, the crosstalk between heart and adipose tissue is significant, and disruption in this crosstalk, particularly in conditions like obesity and heart failure, leads to adverse cardiometabolic outcomes, highlighting the importance of this interaction in maintaining overall health.

## 7. Conclusions

Adipokines are bioactive molecules that are secreted by adipose tissue. Through mediating the communication between adipose tissue and other organs, such as the brain, metabolic tissues, intestines, cardiovascular system, and immune system, they play a critical role in regulating metabolic processes, inflammation, and overall energy balance. This communication is indispensable for the preservation of cardiometabolic health. Obesity, CVD, T2DM, and other metabolic disorders are frequently linked to dysregulated adipokines. Consequently, comprehending the function of adipokines facilitates the development of potential therapeutic interventions for CVD, diabetes, and obesity. Adipokines can also function as biomarkers to evaluate the risk of metabolic and CVDs.

## Figures and Tables

**Table 1:** Adipokines in representative clinical studies.

Study	Population	Comparison	Outcome
Mir et al., 2022 [51]	87 subjects (46 males, 41 females) with T2DM divided into normal BMI, overweight, obese, and severely obese, and 85 healthy controls (44 males and 41 females) Country: The Asir region of Saudi Arabia	Comparing adiponectin, leptin, resistin, visfatin, and chemerin between subjects with T2DM and healthy controls, as well as between different categories of BMI within the T2DM group	Subjects with T2DM had lower adiponectin levels than the control group, with marked reductions in obese and severely obese subjects with T2DM Leptin, visfatin, and chemerin levels were higher in the T2DM group and rose as BMI increased Resistin showed no significant difference between groups
Gradinaru et al., 2017 [64]	83 non-smoking subjects (27 males and 56 females) aged 64–76 years, 44 with metabolic syndrome (MS) and 39 without MS. Country: Romania	Comparing serum adiponectin levels in elderly subjects with and without MS	Elderly subjects with MS had lower levels of adiponectin and higher levels of both oxidative stress and cardiovascular risk markers than those with no MS
Dahl et al., 2007 [89]	21 patients who had an ipsilateral stroke, transient ischemic attack, or amaurosis fugax in the previous 6 months (14 symptomatic plaques and 7 asymptomatic plaques) Country: Norway	Comparing levels of visfatin between patients with symptomatic and asymptomatic plaques	Visfatin expression was higher in patients with symptomatic carotid plaques than in asymptomatic ones
Stengel et al., 2014 [95]	60 hospitalized patients were divided into four groups according to their BMI: normal weight (BMI 18.5–25 kg/m^2^), anorexia nervosa (BMI < 17.5 kg/m^2^), and obesity (BMI 30–40, 40–50, and >50 kg/m^2^, *n* = 15/group) Country: Germany	Comparing DPP4 enzyme activity and its targets, pancreatic polypeptide (PP) and glucagon-like peptide (GLP-1) among the three groups	Obese patients had the highest level of DPP4 concentration and activity PP levels were higher among anorexic patients, compared with normal weight and obese patients No difference between groups in GLP-1 levels
Kovalyova et al., 2017 [116]	83 subjects with essential hypertension were split into two groups according to whether they had hyperglycemia and/or abdominal obesity Country: Ukraine	Comparing serum levels of Nesfatin-1 among hypertensive subjects with and without hyperglycemia and abdominal obesity	Serum Nesfatin-1 levels were elevated in subjects with essential hypertension accompanied by hyperglycemia and/or abdominal obesity in comparison to those with normal blood glucose and body weight
Feng et al., 2023 [121]	128 patients with T2DM (76 males and 52 females) Country: China	Investigate the correlation between serum isthmin-1 levels and HDL-C in patients with T2DM	A significant negative correlation between blood isthmin-1 levels and HDL-C in T2DM (β = −0.235, *p* < 0.001)
Ni et al., 2013 [124]	261 patients (169 men and 92 postmenopausal women; 188 with coronary artery disease (CAD) and 73 without CAD) Country: China	Comparing lipocalin-2 levels in patients with and without CAD and metabolic syndrome	The levels of lipocalin-1 were higher in men than in women, as well as in individuals with CAD and metabolic syndrome compared to those without these conditions
Yan et al., 2018 [167]	311 subjects with T2DM (178 with MS and 133 without MS) Country: China	Comparing neuregulin-4 levels between diabetic patients with and without MS	Plasma neuregulin-4 levels were found to be lower among diabetic patients with metabolic syndrome compared with those without metabolic syndrome
Schories et al., 2023 [191]	450 patients with symptomatic heart disease (177 patients were diagnosed with chronic coronary syndrome, 211 with acute coronary syndrome, and 62 without coronary artery disease) Country: Germany	Comparing platelet surface and plasma-associated CXCL14 levels in patients with chronic or acute coronary syndrome and patients without coronary artery disease	Platelet-associated CXCL14 was lower in patients with chronic coronary syndrome Patients with normal left ventricular ejection fraction had higher levels of CXC14, and vice versa
Ali et al., 2021 [215]	60 obese and 60 non-obese subjects underwent bariatric and hernia repair surgeries, respectively. Country: USA	Analysis of DNA methylation and gene expression of leptin and adiponectin in visceral adipose tissue of obese and lean patients, and its correlation with cardiometabolic risk factors	Leptin showed lower promoter methylation and higher gene expression in the adipose tissue of obese subjects compared to non-obese controls Adiponectin showed higher promoter methylation and lower gene expression in the adipose tissue of obese subjects compared to non-obese controls
Chen et al., 2019 [216]	98 hypertensive patients and 24 normotensive controls Country: China	Comparing the circulating adipokines levels between hypertensive and healthy patients and their relationship to hypertension-related complications.	In hypertensive patients, circulating levels of brain-derived neurotrophic factor were decreased while leptin and irisin levels were increased compared with controls Irisin level was positively correlated with systolic blood pressure and is associated with hypertension-related stroke
Sitticharoon et al., 2014 [217]	35 women, of which 20 are obese and 13 are non-obese Country: Thailand	Correlations between adipokines involved in insulin sensitivity (adiponectin, visfatin, and omentin) and cardiometabolic risk factors in obese and lean patients	Positive correlations between serum adiponectin and serum and subcutaneous omentin-1 and insulin sensitivity index Negative correlations between serum adiponectin and serum omentin-1 and body weight, BMI, and HOMA-IR Negative correlations between serum visfatin and serum omentin-1 and weight gain
